# Tcf12 and NeuroD1 cooperatively drive neuronal migration during cortical development

**DOI:** 10.1242/dev.200250

**Published:** 2022-02-11

**Authors:** Aditi Singh, Arun Mahesh, Florian Noack, Beatriz Cardoso de Toledo, Federico Calegari, Vijay K. Tiwari

**Affiliations:** 1 Wellcome-Wolfson Institute for Experimental Medicine, School of Medicine, Dentistry & Biomedical Science, Queens University Belfast, Belfast BT9 7BL, UK; 2 CRTD-Center for Regenerative Therapies, School of Medicine, Technische Universität Dresden, 01307 Dresden, Germany

**Keywords:** Cortical development, Neurogenesis, Epigenetics, Genomics, Gene regulation, Transcription factors

## Abstract

Corticogenesis consists of a series of synchronised events, including fate transition of cortical progenitors, neuronal migration, specification and connectivity. NeuroD1, a basic helix-loop-helix (bHLH) transcription factor (TF), contributes to all of these events, but how it coordinates these independently is still unknown. Here, we demonstrate that NeuroD1 expression is accompanied by a gain of active chromatin at a large number of genomic loci. Interestingly, transcriptional activation of these loci relied on a high local density of adjacent bHLH TFs motifs, including, predominantly, Tcf12. We found that activity and expression levels of Tcf12 were high in cells with induced levels of NeuroD1 that spanned the transition of cortical progenitors from proliferative to neurogenic divisions. Moreover, Tcf12 forms a complex with NeuroD1 and co-occupies a subset of NeuroD1 target loci. This Tcf12-NeuroD1 cooperativity is essential for gaining active chromatin and targeted expression of genes involved in cell migration. By functional manipulation *in vivo*, we further show that Tcf12 is essential during cortical development for the correct migration of newborn neurons and, hence, for proper cortical lamination.

## Abstract (precis)

**Summary:** How the functional cooperativity of Tcf12 and NeuroD1 in specific subpopulations of the developing cortex creates the gene regulatory program essential for neuronal migration.

## INTRODUCTION

The brain is the most complex organ that has arisen in evolution, but exactly how this complexity is generated during development is still poorly understood. The interplay between signalling molecules, transcription factors (TFs) and epigenetic mechanisms are required to drive the gene expression programs underlying tissue formation. During embryonic development of the mammalian cortex, these programs initially promote neuroepithelial stem cells expansion at the apical boundary of the ventricular zone (VZ) that for this reason are called apical progenitors (APs) (Greig et al., 2013; Paridaen and Huttner, 2014). As development proceeds, changes in transcriptional and epigenetic programs are required to drive an increasing proportion of APs to switch from proliferative to differentiative divisions and generate either basal progenitors (BPs) that leave the VZ to form the subventricular zone (SVZ) or neurons (Basu et al., 2020). Although most APs continue to proliferate, the majority of BPs undergo neurogenic divisions, generating two postmitotic neurons that migrate through the intermediate zone (IZ) to form the cortical plate (CP). Finally, within the CP, a correct cellular specification and layering must ensue for newborn neurons to form proper cortical lamination (Buchsbaum and Cappello, 2019).

Recent research provided many insights into the cellular and molecular mechanisms individually governing each of the many steps required for corticogenesis, including AP expansion, cell fate switch from AP to BP, neurogenesis, neuronal migration and cortical layering (Hu et al., 2012; Lister et al., 2013; Pataskar et al., 2016b). However, the interplay and synergy among epigenetic and gene expression programs executing each of these complex and sequentially interconnected, yet partly independent, processes remain largely unknown.

Basic helix-loop-helix (bHLH) proteins belong to a family of transcription factors (TFs) that are crucial for many developmental processes. Typically, bHLH TFs form homo- or hetero-dimers with other bHLH proteins, which accounts for their DNA-binding features (Boeva, 2016). These dimers bind E-box sequence motifs to regulate cell renewal and lineage specification (Le Dréau et al., 2018; Bertrand et al., 2002). Previously, our group has demonstrated that NeuroD1, a bHLH TF, functions as a pioneer transcription factor (PTF) by binding heterochromatic regions at neuronal genes and subsequently imposing a euchromatic state (Pataskar et al., 2016a). In turn, this allows downstream TF binding and regulation of gene expression to promote neurogenesis. NeuroD1 is a class II bHLH TF that is active in heterodimeric forms (Naya et al., 1995; Poulin et al., 2000). In addition to its role in the transition from AP to BP and neurogenesis, NeuroD1 is also essential for migration as well as maturation and survival of newborn neurons (Boutin et al., 2010; Choi et al., 2020; Gao et al., 2009; Aprea et al., 2014). Given the need to precisely coordinate all of these complex events occurring in different cell types and at different times, it is puzzling that a single PTF, NeuroD1, alone can influence them all. In more general terms, how individual bHLH factors, PTFs or TFs are sequentially regulated across cell types to act on different cellular events and mechanisms remains largely unexplored. NeuroD1 has been reported to form heterodimer complexes with class I bHLH factor E47 (Del Olmo Toledo et al., 2018). Building upon these previous observations and the known biology of bHLH TFs, we speculated that NeuroD1 could potentially interact with other distinct bHLH factors to govern different processes underlying corticogenesis.

Here, we find that NeuroD1 bound target sites exhibit distinct DNA shape features and motif enrichment for other bHLH TFs, including Tcf12. We show that Tcf12 is co-induced and interacts with NeuroD1 to form a heterodimer, and co-occupies a specific subset of NeuroD1 target genomic loci at the onset of neurogenesis during cortical development. Moreover, we show that such cooperativity is essential for a gain in active chromatin and induction of neuronal migration genes. Finally, downregulation of Tcf12 during cortical development specifically impairs neuronal migration. Together, our findings highlight the potential of NeuroD1 to synchronise multiple events during corticogenesis in different cell types by its modular pairing with distinct bHLH TFs.

## RESULTS

### Genetic features govern the differential transcriptional response of NeuroD1 during neurogenesis

**Fig. 1.**
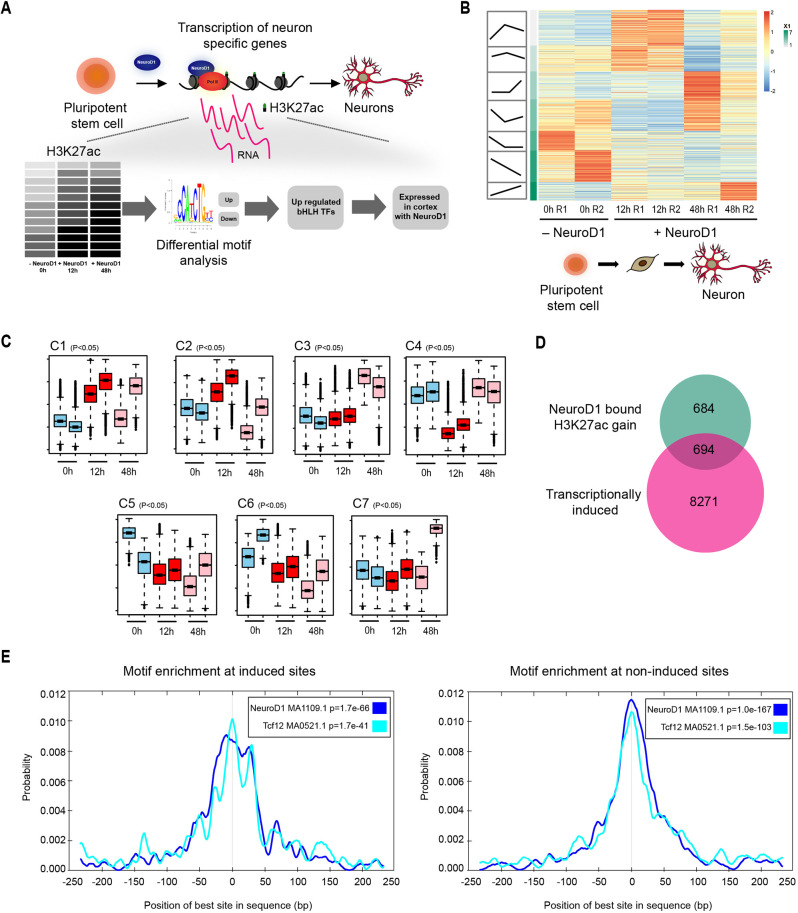
**Dynamics of the H3K27ac mark following NeuroD1 expression.** (A) Schematic of analysing H3K27ac dynamics upon NeuroD1 expression. (B) Global dynamics of H3K27ac enrichment before and after NeuroD1 induction (12 h and 48 h). R1 and R2 are replicates. (C) Boxplots show average H3K27ac enrichment in each cluster (C1 to C7). Middle bars show median values, boxes show first to third interquartile ranges, whiskers show the minimum and maximum of data range and beyond that are outliers. (D) NeuroD1-bound and transcriptionally induced sites. (E) Motif enrichment profiles at transcriptionally active and inactive sites.

We have previously established a system whereby NeuroD1 is selectively induced in mouse embryonic stem cells, triggering their differentiation. These differentiated cells acquire both molecular and phenotypic hallmarks of neurons within 48 h of NeuroD1 expression (Pataskar et al., 2016a). Using this system, we demonstrated that NeuroD1 functions as a PTF to remove repressive chromatin marks, and induce chromatin remodelling and activation of the target neuronal genes (Pataskar et al., 2016a). Here, we employed this approach to comprehensively investigate genome-wide chromatin remodelling in response to NeuroD1 expression during neurogenesis. To achieve this, we performed chromatin immunoprecipitation (ChIP) assays for H3K27ac, 12 and 48 h after NeuroD1 induction, followed by high-throughput sequencing (ChIP-seq) (Fig. 1A). This showed the differential dynamics of H3K27ac enrichment at various genomic regions during differentiation (cluster 1-7 in Fig. 1B,C). Similar observations were made at NeuroD1 target sites, as derived from the ChIP-seq assay for NeuroD1 after 48 h of induction (Fig. S1A). For example, some sites continuously gained H3K27ac over time, whereas other genomic sites gained acetylation at 12 h and then lost it. Such dynamic reprogramming of H3K27ac levels at gene regulatory regions was consistent with previous reports and shown to be associated with cell-fate switches during development (Bogdanović et al., 2012; Tiwari et al., 2018; Thakurela et al., 2015).

We next attempted to correlate NeuroD1 occupancy (ChIP-seq) and changes in H3K27ac (ChIP-seq) at its target sites with changes in gene expression (RNA-seq). Interestingly, although a large number of NeuroD1-bound loci gained active chromatin (1378), only approximately half of these were transcriptionally induced (694) (Fig. 1D). Intrigued by this differential transcriptional behaviour despite NeuroD1 targeting and a gain in active chromatin, we next investigated whether any genetic features were associated with these differences. Specifically, a motif enrichment analysis revealed that the top 10 enriched motifs were identical at induced and non-induced sites, with the exception of only one, Tcf12, which was enriched exclusively at the induced sites (Fig. S1B). Interestingly, these top enriched motifs entirely represented various bHLH TFs (Fig. S1B). Furthermore, at the induced sites, NeuroD1 and Tcf12 motifs were enriched at a much higher frequency adjacent to the peak centre when compared with the non-induced sites, where the intensity is highest only at the centre (Fig. 1E). The presence of repeated motif patterns provides the accessory binding sites for TFs and may work as the shadow sites to the original transcription factor binding site. The role of shadow sites has been increasingly explored in recent studies to support TF binding at promoters as well as at enhancer clusters (Papatsenko et al., 2002; Kozlov et al., 2015; Boeva, 2016).

The differential enrichment and motif density distribution at NeuroD1 target sites prompted us to further investigate their biophysical features. DNA shape features are known to determine the gene regulatory potential of genomic loci (Pataskar et al., 2019), e.g. through regulating the DNA-binding affinity of TFs (Samee et al., 2019). In some cases, DNA shape features allow two different TFs to bind at the same region, even though they do not physically interact with each other (Ibarra et al., 2020). Analysis of different biophysical attributes at these genomic regions using DNAShapeR (Chiu et al., 2016) revealed that the induced sites exhibited a higher minor groove width (MGW) and propeller twist (ProT) in comparison with the non-induced sites (Fig. S1C). Interestingly, similar to motif intensity, these features also had increasing magnitude/expanse not just at the centre but also at the adjacent loci for induced sites. These features are thought to confer an increased surface area and facilitate accommodating co-transcription factors (Pataskar et al., 2019), consistent with the co-occupancy of NeuroD1 and Tcf12 at the transcriptionally active sites.

### Tcf12 is a potential co-factor of NeuroD1 during cortical development

**Fig. 2.**
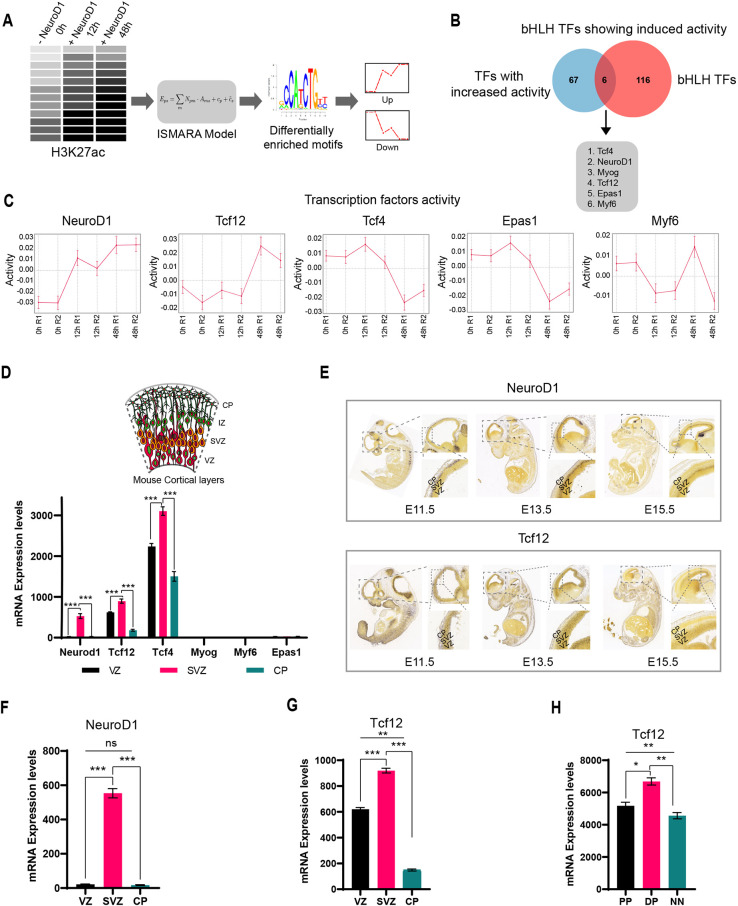
**Identification of potential NeuroD1 co-factors.** (A) Schematic of ISMARA analysis for co-enriched TF selection. (B) Overlap of upregulated motifs predicted from ISMARA with bHLH transcription factors. (C) Activity profiles for selected motifs from the overlap of ISMARA motifs and bHLH factors. R1 and R2 are the replicates for each time point (0 h, 12 h and 48 h). Data are mean±s.d. (D) Expression of selected factors in different cortical layers. (E) *In situ* hybridisation images (Allen Brain Atlas) showing the expression of NeuroD1 and Tcf12 in developing cortex at E11.5, E13.5 and E15.5. (F) Bar chart showing the expression level of NeuroD1 in VZ, SVZ and CP regions. (G) Bar chart showing the expression level of Tcf12 in VZ, SVZ and CP regions. (H) Bar chart showing the expression level of Tcf12 in proliferating progenitors (PP), differentiating progenitors (DPs) and neurons (NNs). Data are mean±s.d. Statistical significance was calculated using a paired two-tailed Student's *t*-test. *P<0.05, **P<0.01, ***P<0.001.

Given that bHLH TFs function only as hetero- or homodimers, and NeuroD1 does not bind to E-box motifs as a homodimer (Naya et al., 1995; Ray and Leiter, 2007), we hypothesised that NeuroD1 and Tcf12 potentially form heterodimers to control gene expression during neurogenesis. To substantiate our hypothesis, we employed ISMARA (integrated motif activity response analysis) that uses gene expression or chromatin state data across a set of samples to identify key TFs driving the observed expression/chromatin state changes (Balwierz et al., 2014). ISMARA analysis was performed for H3K27ac ChIP-seq data derived at 0, 12 and 48 h following NeuroD1 induction (Fig. 2A). The TFs that showed significantly increased activity during the time-course overlapped with genes that were upregulated at 48 h after NeuroD1 induction (Fig. S2A). Further analysis of these genes identified six bHLH TFs that showed induced activity at H3K27ac sites and were transcriptionally active during NeuroD1-induced neurogenesis (Fig. 2B,C). Tcf12 showed similar activity and was predicted to be enriched together with Myog, but these are two independent TFs that exhibit identical motifs. However, as Myog is not expressed in the cortex, only Tcf12 was further pursued as a relevant TF (Fig. S2C,D). We also performed ISMARA analysis in our RNA-seq timecourse data and observed activity for the same and other TFs (Fig. S2B). Gene expression analysis of these six TFs in the developing cortical layers showed that only Tcf12, Tcf4 and NeuroD1 were expressed in the E14 embryonic cortex and induced in the SVZ layer compared with the VZ layer (Fig. 2D).

Using existing data of single-cell RNA-sequencing at high temporal resolution that tracks the lineage of the molecular identities of successive generations of neural progenitors and neurons in mouse embryos (Telley et al., 2016), we observed Tcf12 induction together with NeuroD1 as cells transit from an AP to a BP state (Fig. S2C). A pseudo-timecourse analysis further validated the simultaneous upregulation of NeuroD1 and Tcf12 during neurogenic commitment and their gradual downregulation in newborn neurons (Fig. S2D). Next, we analysed the expression of NeuroD1 and Tcf12 in the developing brain from three different developmental stages (E11.5, E13.5 and E15.5) using the Allen Brain atlas database (Thompson et al., 2014) and we observed that, at E13.5, both NeuroD1 and Tcf12 expression were highly enriched in the SVZ (Fig. 2E). Similar results were obtained from E14.5 RNA-seq data, where both NeuroD1 and Tcf12 were highly enriched in the SVZ relative to the VZ and IZ/CP (Fig. 2F,G). We next analysed the expression of Tcf12 in specific populations of proliferating progenitors, differentiating progenitors and newborn neurons, as defined in an earlier study by our lab (Aprea et al., 2013), and found that Tcf12 was highly expressed in differentiating progenitors (DPs) compared with proliferating progenitors (PP) and differentiated neurons (N) (Fig. 2H). Unfortunately, we could not confirm the expression kinetics of Tcf12 at the protein level as the commercially available antibody consistently gave a poor signal in immunohistochemistry despite attempts under various conditions. To circumvent this and strengthen our observations, we further analysed a publicly available scRNA-seq data that revealed that cells expressing Tcf12 and NeuroD1 begin to co-express early-born postmitotic neuronal markers, such as Tbr1 (Fig. S2D) (Telley et al., 2016). These observations are consistent with our earlier hypothesis that NeuroD1 and Tcf12 act together to contribute to neurogenesis during cortical development.

**Fig. 3.**
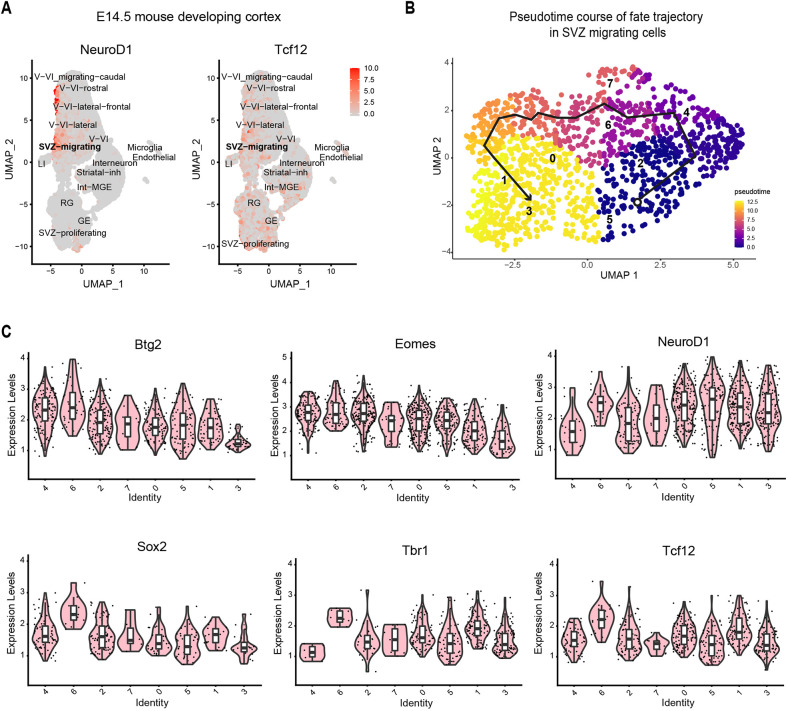
**NeuroD1 and Tcf12 are co-expressed in distinct subpopulations during cortical development.** (A) Expression of NeuroD1 and Tcf12 in different brain cell types in E14.5 cortex shows the highest NeuroD1 expression in the migrating neuronal population and the Tcf12 expression levels. (B) Pseudo-timecourse trajectory of SVZ migrating cells. Numbers show the clusters arranged in developmental pseudotime. (C) Expression of progenitor, neurogenic and neuronal markers in a SVZ migrating subpopulation arranged by pseudotime, where we see increasing NeuroD1 expression levels.

To further investigate the co-regulatory function of NeuroD1 and Tcf12 during cortical development, we comprehensively analysed an independent high-quality single-cell transcriptome dataset from E14.5 cortex (Loo et al., 2019) and performed clustering and cell-type annotations as previously described (Loo et al., 2019) (Fig. S2E). Interestingly, SVZ migrating population was found to express highest levels of NeuroD1 along with the layer V-VI migrating neurons, and both cell types were also found to express high levels of Tcf12 (Fig. 3A). Intriguingly, Tcf12, but not NeuroD1, was also highly expressed in SVZ proliferating and endothelial cells, suggesting additional NeuroD1-independent functions for Tcf12.

Following up on our earlier observations, we focussed on SVZ migrating cell populations and further classified these into eight subpopulations that show distinct NeuroD1 expression levels (Fig. S2F). Thereafter, the pseudo-timecourse analysis for the subpopulations in which the root population (cluster 4) contained the lowest levels of neuronal markers, organised them into a lineage trajectory developing towards neuronal fate (Fig. 3B,C). Further analysis using various progenitor, neurogenic and neuronal markers showed these populations gradually progressing towards a neurogenic fate. For example, we observed that earlier clusters in the lineage trajectory expressed low levels of NeuroD1 and higher expression of progenitor marker (Sox2, Nes and Vim) expression, whereas subsequent clusters expressed increasing levels of NeuroD1 and higher levels of neuronal markers (Tbr1, Tubb3 and Tuba1a), while simultaneously decreasing differentiating progenitor (Eomes and Btg2) markers (Fig. 3B, Fig. S3A). Although the earlier SVZ migrating clusters express proliferating markers, the levels are comparatively much lower than in progenitor cell populations in the E14.5 cortex (Fig. S4). In line with our previous observations, such gradual expression of NeuroD1 and gain of neuronal identity further accompanied a progressive gain in Tcf12 expression (Fig. 3B). In addition to Tcf12, several other bHLH factors were also expressed in distinct SVZ subpopulations and warrant further investigation (Fig. S3B).

TF networks and their conserved co-expression from rodents to primates are helpful to yield fundamental insights into mammalian/human biology (Cheng et al., 2014; Pembroke et al., 2021; Bogenpohl et al., 2019). To broaden our understanding of such co-regulated genomic sites in cortical development, we analysed genes characterised by NeuroD1 binding, Tcf12 motif intensity, enrichment of H3K27ac and transcriptionally induced in E14.5 mouse cortex, and found that the majority of them were induced upon neurogenesis (Fig. S5A,B). In line with these observations, the majority of these loci showed similar transcriptional behaviour in the developing human cortex, suggesting that the NeuroD1-Tcf12 network may have a similar function in the developing human cortex (Fig. S5C).

### Tcf12 interacts with NeuroD1 and co-occupies NeuroD1 target sites to induce active chromatin

**Fig. 4.**
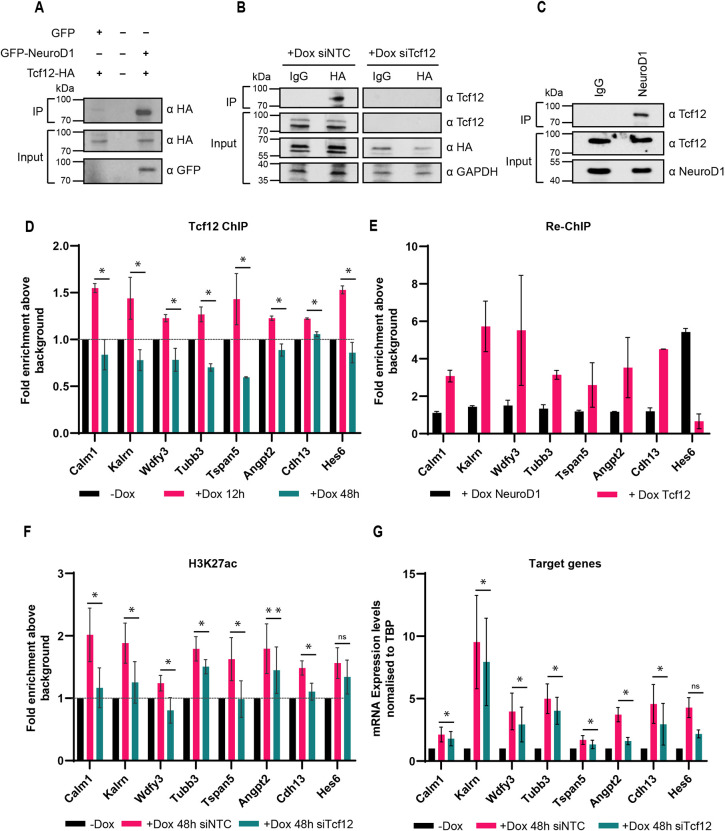
**Tcf12-NeuroD1 complex induces active chromatin and expression of neuronal migration genes.** (A) Co-IP experiment showing GFP-tagged NeuroD1 interacting with HA-tagged Tcf12 *in vitro*. This specific interaction was absent in control immunoprecipitates where we co-expressed HA-tagged Tcf12 with only GFP protein. (B) IP experiment showing the specific enrichment of endogenous Tcf12 by NeuroD1 only in the negative control siRNA (left panel). It was absent in Tcf12 knockdown conditions (right panel) during *in vitro* neurogenesis. (C) IP experiment from the E14.5 mouse cortex showing specific interaction of Tcf12 with NeuroD1 during cortical development *in vivo*. (D) ChIP qPCR results showing the enrichment of Tcf12 at NeuroD1 target sites after 12 h and 48 h of NeuroD1 induction. (E) ChIP Re-ChIP qPCR experiment showing the NeuroD1 and Tcf12 co-binding at the same target genes. (F) qPCR results showing a change in H3K27ac levels at NeuroD1 target sites upon Tcf12 knockdown. (G) qPCR results showing the expression of NeuroD1 target genes upon Tcf12 knockdown. All the ChIP experiments were performed as three independent biological replicates. The ChIP-Re-ChIP experiment was repeated twice. Data are mean±s.d. Statistical significance was calculated using a paired two-tailed Student's *t*-test. *P<0.05, **P<0.01.

Based on our findings, we speculated that NeuroD1 may form a heterodimer with Tcf12 in specific subpopulations during cortical development. In line with this hypothesis, we next sought to validate the physical interaction of NeuroD1 and Tcf12. Using BioGrid (Stark et al., 2006), a protein-protein interaction network database, we found that Tcf12 was among the proteins most significantly associated with NeuroD1 in affinity capture assays (Fig. S6A). Prompted by this, we performed immunoprecipitation assays using lysates of P19 cells by co-expressing GFP-tagged NeuroD1 and HA-tagged Tcf12. We found that NeuroD1 efficiently co-immunoprecipitated Tcf12 (Fig. 4A), consistent with their formation of complexes. Validating this conclusion and the specificity of binding, a siRNA-mediated depletion of Tcf12 led to its loss in the blot (Fig. 4B).

To confirm the interaction of NeuroD1 with Tcf12 *in vivo*, we first assessed our ability to detect both proteins when expressed at physiological levels by performing western blot analysis of lysates from the E14.5 cortex (Fig. S6B). Next, we performed immunoprecipitation for NeuroD1 and confirmed the detection of Tcf12 in the resulting precipitates (Fig. 4C), in turn validating the physical interaction of both TFs in the developing cortex.

We next investigated whether Tcf12 is recruited to NeuroD1 target sites. An analysis of the accessibility of NeuroD1 binding sites in proliferating, differentiating and neuronal cell populations (Aprea et al., 2013) showed consistently high activity for Tcf12 at these loci in differentiating and neuronal populations, in line with our earlier observations of its potential active chromatin inducing activity (Fig. S6C). We next performed ChIP assays for Tcf12 during NeuroD1-induced neurogenesis and tested NeuroD1 target regions for Tcf12 occupancy using quantitative PCRs. Target and non-target genes were selected for being NeuroD1 targets, increased H3K27ac enrichment and expression at 48 h with and without the occurrence of Tcf12 motifs. These Tcf12 and NeuroD1 co-occupied genes included established neuronal migration genes, such as Wdfy3, Calm1, Kalrn and Angpt2. This analysis showed that, indeed, Tcf12 co-occupies NeuroD1-binding sites during *in vitro* neurogenesis (Fig. 4D). To further validate the co-occupancy, we performed NeuroD1 ChIP followed by Tcf12 re-ChIP and quantified their co-occupancy at the target sites by qPCRs. These results showed that indeed Tcf12 and NeuroD1 co-occupy these target genomic regions (Fig. 4E). When comparing the dynamic binding behaviour of Tcf12 over time, at 12 h we observed an increased enrichment at its target genes relative to that at 48 h, when we observed a significant reduction in binding (Fig. 4F,G). This is consistent with our earlier observations and suggests that as progenitors switch to neurogenesis, Tcf12 is recruited at its target sites to induce their expression by changing their chromatin landscape and then leaves these sites as newborn neurons mature.

We next assessed whether Tcf12 is essential to induce an active chromatin mark (H3K27ac) and gene activation at NeuroD1 target sites during neurogenesis. For this, we performed Tcf12 knockdown during NeuroD1-induced neurogenesis using siRNAs against Tcf12 and quantified the H3K27ac levels at NeuroD1 target sites by ChIP-qPCR. Interestingly, in the absence of Tcf12, the NeuroD1 target sites showed reduced levels of H3K27ac (Fig. 4F), which was accompanied by a reduction in the expression of the corresponding genes (Fig. 4G). These findings were confirmed by visualisation of peak enrichment in genomic browser for individual genes (e.g. Nhlh1) from the genome-wide datasets (Fig. S6D). We confirmed the validity of Tcf12 targeting only a subset of NeuroD1 target sites by performing qPCR analysis for several control genes to show lack of Tcf12 motif enrichment (Fig. S7A), the targeting of NeuroD1 (Fig. S7B), a gain in active chromatin (Fig. S7C) and expression of these NeuroD1 target genes (Fig. S7D) during neurogenesis. Altogether, we conclude that Tcf12 interacts with NeuroD1 and co-occupies distinct loci to drive the induction of active epigenetic state and gene expression program underlying neurogenesis.

### Loss of Tcf12 during cortical development impairs neuronal migration

**Fig. 5.**
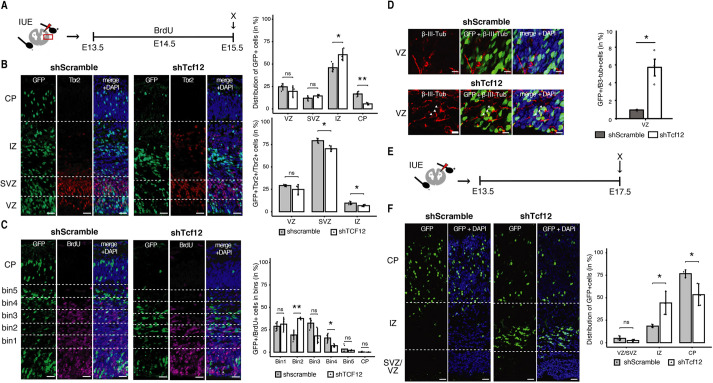
**Loss of Tcf12 impairs neuronal migration during cortical development.** (A) Experimental layout for the *in utero* electroporation (IUE) assay where IUE was carried out at E13.5 and analysed at E15.5. (B) Immunofluorescence images showing phenotypic changes following depletion of Tcf12. Here, E15.5 cortex electroporated at E13.5 with a control shscramble or a shTcf12 plasmid containing a GFP reporter (left panel) was analysed for the distribution of GFP^+^ and GFP^+^ Tbr2^+^/Tbr2^+^ cells (in %) across the cortical layers (right panel). (C) Neuronal migration was assessed by identifying five equidistant bins across the SVZ/IZ (left panel) and quantifying the distribution of GFP^+^/BrDU^+^ population (in %) in these bins (right panel). (D) Immunofluorescence images showing the expression of the neuron-specific marker β-III-Tubulin upon Tcf12 knockdown (left panel) and the distribution of GFP^+^/β-III-Tub^+^ cells (in %) in the ventricular zone (VZ) (right panel). (E) Experimental schematic for the *in utero* electroporation (IUE) assay where IUE was carried out at E13.5 and analysed at E17.5. (F) Immunofluorescence images showing phenotypic changes in the developing cortex following a prolonged knockdown of Tcf12 (left panel). The distribution of electroporated cortical cells (GFP^+^ cells) across the cortical layers is shown (in %) (right panel). Scale bars: 100 µm. Statistical significance was calculated using an unpaired two-tailed Student's *t*-test. *P<0.05, **P<0.01; *n*=3 or 4 independent biological replicates. Data are mean±s.d.

We next investigated the role of Tcf12 during *in vivo* cortical development. To achieve this, we performed *in utero* electroporation to deliver plasmid vectors encoding a shRNA targeting Tcf12 or a scramble sequence as a control. To begin with, electroporation was performed at E13.5, as previously described (Aprea et al., 2014; Pataskar et al., 2016a), and brains were harvested 48 h later (Fig. 5A). Immunohistochemistry analysis revealed that the proportion of targeted cells and their progeny identified by a GFP reporter co-expressed together with the scramble or shRNAs sequences was unaffected within the proliferative layers of the VZ (24.6 versus 19.6%; *P*=0.29) and SVZ (11.9 versus 14.4%; *P*=0.17). In contrast, shTcf12-targeted brains displayed an increase in GFP^+^ cells within the IZ (45.9 versus 60.4%; *P*=0.04) that was accompanied by the almost complete depletion of GFP^+^ cells in the CP (16.6 versus 5.6%; *P*=0.001) (Fig. 5B).

Next, we investigated whether the reduction in GFP^+^ cells within the CP after depletion of Tcf12 was due to impairments in the switch of neuronal progenitors from proliferation to neurogenesis. To do this, we used the intermediate progenitor marker Tbr2 to distinguish AP, BP and neurons as (1) Tbr2^–^ cells within the VZ, (2) Tbr2+ cells in the VZ or SVZ, and (3) cells in the IZ/CP irrespective of Tbr2, respectively. This revealed that the proportion of the three cell types across the cortex (14.7 versus 16.6%, *P*=0.35; 25.4 versus 26.2%, *P*=0.78; and 68.0 versus 66.8%, *P*=0.60, respectively) was not significantly affected by Tcf12 depletion. However, interestingly, although the total number of BP across all cortical layers did not significantly change upon Tcf12 knockdown, we observed that their distribution was unbalanced with a minor, yet significant, decrease specifically within the SVZ/IZ (79.2 versus 70.2%; *P*=0.04; 9.5 versus 6.7%; *P*=0.01 for the SVZ and IZ, respectively, Fig. 5B). This, in turn, was consistent with our previous results in neuronal cell cultures (Fig. S7E), suggesting that Tcf12 is not essential for cell identity and fate commitment to neurogenesis but likely is important for migration.

To explain the imbalance in the proportion of BP located within the SVZ/IZ and increase in GFP^+^ cells in the IZ at the expense of those in the CP upon Tcf12 depletion (Fig. 5B), we next assessed neuronal migration by specifically birthdating newborn neurons with a single administration of BrdU at E14.5, i.e. 24 h after delivery of control or shRNA against Tcf12. One day later at E15.5, BrdU^+^ cells were quantified in equidistant bins across the SVZ/IZ, revealing their increased accumulation at the boundary between SVZ and IZ upon Tcf12 knockdown (bin 2: 19.2 versus 37.6%; *P*=0.005). Concomitantly, the proportion of GFP^+^ BrdU^+^ cells within the bins 3 to 5 were reduced (32.4 versus 18.2%; *P*=0.08; 15.8 versus 7.2%; *P*=0.03 for bin 3 and 4, respectively) (Fig. 5C). Furthermore, again confirming the effect of Tcf12 knockdown on neuronal migration, inspection within the VZ after immunohistochemistry for the neuronal marker β-III-tubulin revealed a sixfold increase in newborn neurons among GFP^+^ electroporated cells and their progeny relative to controls (0.9 versus 5.7%; *P*=0.0059) (Fig. 5D), which is most likely the result of an impaired migration of neurons resulting from direct neurogenesis from APs.

Finally, to investigate whether or not effects on neuronal migration were transient, we repeated similar experiments upon electroporation with scramble or Tcf12-shRNAs at E13.5 as before, but this time analysing brains 4 days later at E17.5 (Fig. 5E). Even more than before, the distribution of GFP^+^ cells was severely affected, with nearly all GFP^+^ cells being located within the CP in controls and, conversely, the overwhelming majority being retained within the IZ in shRNA-targeted brains (Fig. 5F). Highlighting an almost complete impairment in neuronal migration both at 2 and 4 days after knockdown, our data extend to physiological development the reported role of Tcf12 in the migration of tumour cells (Poulin et al., 2000; Mesman and Smidt, 2017).

## DISCUSSION

Understanding the mechanisms underlying cell fate commitment is key to decipher the fundamental biological processes driving tissue formation during development and regeneration in disease. The cell-fate specification requires an intricate interplay between signalling pathways, DNA-binding TFs and epigenetic machinery. In this study, we investigated how a pro-neural pioneer TF, NeuroD1, functions at a genome-wide level to induce active chromatin and gene expression, and coordinate specific cellular events during corticogenesis through its interaction with another bHLH TF: Tcf12.

Histone modifications regulate chromatin accessibility to control transcription. Histone acetylation at lysine 27 is a mark of active enhancers and promoters (Creyghton et al., 2010). We discovered that although NeuroD1 targets a large number of genomic loci that gain active chromatin (H3K27ac), only a subset of them are transcriptionally induced. Discriminating transcriptionally active and inactive sites revealed their unique profiles of motif occurrences. We found that induced sites form a high-affinity platform for TF binding, with a higher number of motifs and providing accessory binding sites at which other TFs can bind together with NeuroD1 and co-regulate gene expression. These accessory sites are low-affinity shadow sites for TF binding that themselves cannot retain TF proteins for gene regulatory processes, but increase TF concentration at the TF binding domains (Kozlov et al., 2015; Boeva, 2016). bHLH factors recognise a common consensus (CANNTG) motif and have specificity for sequences around it and these specific, or sometimes called ‘private’, binding sites are associated with lineage-specific gene transcription (Fong et al., 2015). Such attributes are helpful to recruit diverse factors at certain genomic loci with changing transcriptional environments. Therefore, lineage-determining factors can interact with different E-box proteins and recruit them to specific target sites. Some studies emphasise the role of these sites for TF recruitment and lateral diffusion of TF onto the strong affinity sequences. Specifically, the repeated motif occurrences are known to form clusters in enhancer regions and facilitate strong TF binding. These accessory motif instances around the TF-binding sites underlie co-operative DNA binding (Chronis et al., 2017) and co-factor-based regulation of these targets by sequence-specific TFs. The heterogeneity in the E-box motif that is bound by different bHLH dimers determines diverse functions (Le Dréau et al., 2018) during various stages of development (Fairman et al., 1993; Ishii et al., 2012; Johnson, 2020).

Our motif activity analysis of NeuroD1-mediated active sites revealed that Tcf12 was highly enriched at induced sites that showed expression kinetics similar to NeuroD1 during cortical development. Interestingly, NeuroD1 and Tcf12 motifs were enriched at much higher frequency adjacent to the peak centre, providing accessory-binding sites for TFs when compared with the non-induced sites that show only centred motif enrichment that potentially makes them less likely to be bound by active TF dimers. Tcf12 has been shown to have varying roles in different tissues for cell differentiation, migration (Mesman and Smidt, 2017; Mesman et al., 2018; Li et al., 2017; Yoon et al., 2015; He et al., 2016), as well as developmental malformations (Sharma et al., 2013). Given these diversified roles and being an E family protein, it provided a particularly strong candidate as a co-regulator of NeuroD1, which is a lineage-determining factor. Tcf12 expression co-existed within high NeuroD1-expressing SVZ migrating cells. Further dissecting the subpopulation of these SVZ cells revealed that increasing Tcf12 and NeuroD1 expression accompanied a gradual acquisition of neuronal fate.

Our study shows that Tcf12 heterodimerise with NeuroD1 during neurogenesis and cortical development. This pairing was accompanied with Tcf12 co-occupancy of NeuroD1 target sites relevant for nervous system development, including differentiation, axonogenesis and axon guidance. Our analysis of Tcf12-binding kinetics revealed the dynamics of Tcf12 binding at NeuroD1 target sites where Tcf12 enrichment was highest in the early stages of neurogenic commitment and reduced as neuronal maturation proceeded. From our data, it is conceivable to conclude that Tcf12 is required to set active chromatin and initiate transcription at NeuroD1 target sites, but is no longer required at later stages. In support of this, depletion of Tcf12 caused a reduction in active chromatin levels at these co-bound loci, which substantiate our conclusion that cooperativity of NeuroD1 and Tcf12 is responsible for the epigenomic remodelling that drives the gene regulatory program underlying neurogenesis. Notably, the targets of Tcf12 include established neuronal migration genes that failed to gain active chromatin and be transcribed in the absence of Tcf12 during neurogenesis. Our results refine the role of NeuroD1 in neuronal migration specifically through its co-operativity with Tcf12. In line with this, depletion of Tcf12 in the developing mouse cortex specifically impaired neuronal migration without affecting the progenitors themselves or their generation of newborn neurons, which extends previous observations on the migration of tumour cells (Mesman and Smidt, 2017; Poulin et al., 2000) to an essential role in brain development. As Tcf12 is also expressed in other cortical cells that do not have NeuroD1, it would be interesting to investigate its other functions during cortical development.

**Fig. 6.**
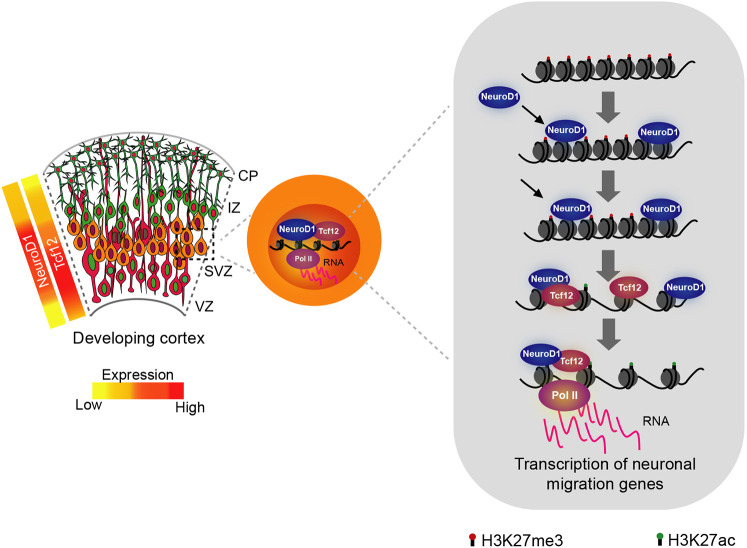
**A schematic showing how the functional cooperativity of Tcf12 and NeuroD1 in specific subpopulations of the developing cortex creates the gene regulatory program essential for neuronal migration.** Both Tcf12 and NeuroD1 are highly co-expressed during the transition of cortical progenitors from proliferative to neurogenic divisions. During this phase, Tcf12 forms a complex with NeuroD1 and co-occupies a subset of NeuroD1 target loci. This Tcf12-NeuroD1 cooperativity is essential for a gain in active chromatin and expression of neuronal migration genes, and therefore for the correct migration of newborn neurons.

Taken together, our findings provide novel insights into the mechanisms underlying gene regulatory programs controlled by NeuroD1 via its combinatorial interaction with other bHLH TFs that orchestrate specific sequential cellular events during corticogenesis. This study identified one such bHLH TF, Tcf12, that cooperates with NeuroD1 to activate epigenetic programs underlying neuronal migration (Fig. 6). Our data show that only a subset of distinct NeuroD1 targets sites are co-occupied by Tcf12, which suggests that Tcf12 might not be the only factor relevant for the NeuroD1-mediated neuronal program. Given its fate-determining potential, NeuroD1 may partner with other bHLH factors (Fischer et al., 2014) at other target sites and cell subpopulations to regulate different aspects of cortical development (Pollak et al., 2013) and neuronal subtype differentiation (Earley et al., 2021). In addition, similar to Tcf12, NeuroD1 induces the expression of other partner bHLH factors, and future studies should investigate their co-regulated genomic targets and their potential role in cortical development. Furthermore, we found that the expression of a set of other bHLH factors correlates or anti-correlates with NeuroD1 in distinct subpopulations during cortical development. Many of these TFs are transcriptional repressors or activators, inhibitors of bHLH dimer formation as well as a component of chromatin remodelling complexes; hence, they are interesting candidates for future investigation. NeuroD1 overexpression has shown promises in regenerative therapy following spinal cord injury and sciatic nerve injuries (Puls et al., 2020; Lai et al., 2020). Given that NeuroD1 partners with other bHLH TFs, discovering such partner TFs may increase success in efforts towards regenerative medicine.

These observations also support the importance of future studies on the cascades of TF activation and their cooperativity during cortical development using recent cutting-edge genomics and proteomics approaches, and they reveal the underlying transcriptional regulatory networks. Promoting new directions of investigation, our study proposes a novel molecular mechanism whereby single PTFs play multiple functions by sequentially co-operating with distinct TFs in a modular and combinatorial way. More studies are needed to investigate this model, which may extend beyond brain development and emerge to be relevant in other organ systems, adulthood and disease.

## MATERIALS AND METHODS

### Cell culture

Cells were cultured as described previously (Pataskar et al., 2016a). Briefly, A2Lox murine ES cells were cultured at 37°C in 7% CO_2_ in 8 ml of ES medium [DMEM supplemented with 10% foetal calf serum, 1× MEM NEAA, 2 mM l-glutamine and LIF on feeders (inactivated MEFs)]. For experiments, the feeders were removed by splitting the ES cells every 2 days onto tissue culture dishes coated with 0.2% gelatin, and the medium was changed daily. Experiments were performed after five passages of the feeder-free state. Transgenic A2lox ES (A2lox-NeuroD1) cells harbouring the murine NeuroD1 CDS (NM_010894.2) fused to an N-terminal HA-tag under the control of a doxycycline-inducible promoter were generated according to Iacovino et al. (2011). Ectopic induction of NeuroD1 was achieved with 500 ng/ml doxycycline for indicated durations. Murine embryonic carcinoma P19 cells were cultured at 37°C in 5% CO_2_ and 88% relative humidity in MEM-α, with 10% newborn foetal calf serum and 1% penicillin/streptomycin. Animal experiments were approved by the local authority Landesdirektion Sachsen (TVV 13/2016 TVV and 16-2018).

### Co-immunoprecipitation

Co-immunoprecipitation (Co-IP) was performed as mentioned earlier (Mahesh et al., 2020). Briefly for the Co-IP, P19 cells were co-transfected with pcDNA4-HA-Tcf12 with either pEGFP-C1 vector alone as control or pEGFP-NeuroD1. At 48 h of post-transfection, cells were washed with PBS and collected. Cells were lysed in lysis buffer [10 mM Tris (pH 7.5), 150 mM NaCl, 0.1 mM EDTA, 0.5% NP-40 and protease inhibitor cocktail (Roche)] and the lysates were diluted with dilution buffer [10 mM Tris (pH 7.5), 150 mM NaCl and 0.1 mM EDTA] to bring to a 0.1% final concentration of NP40. For the IP, cell lysates were incubated with 25 µl of GFP-trap beads (Chromotek) for 8 h at 4°C with rotation. After incubation, beads were washed trice with wash buffer [10 mM Tris (pH 7.5), 150 mM NaCl, 0.5 mM EDTA and 0.1% NP-40] and boiled with 2×SDS loading buffer for 5 min for elution. The eluted fractions were immunoblotted with HA (Abcam ab9110) antibody (1:1000) or GFP (SCBT sc-9996) (1:1000) antibody.

For the endogenous IP, NeuroD1 expression was induced in Transgenic A2lox ES cells by adding doxycycline. After 48 h of NeuroD1 induction, cells were lysed as mentioned earlier. Precleared lysates (100 μg) were taken per IP and incubated with 5 µg of anti-HA antibody (Abcam ab9110) and normal IgG (CST 2729S) antibody for overnight at 4°C with rotation. Protein-A beads (40 μl) were added to the lysates and incubated for 3 h at 4°C with rotation. After the washing, beads were boiled with SDS loading buffer for 5 min for elution. The eluted fractions were immunoblotted with Tcf12 antibody (SCBT sc-28364X) (1:1000).

### Immunoprecipitation from mouse embryonic cortex

Cortex was dissected from C57BL/6J mouse E14.5 embryos and lysed with gentle lysis buffer [10 mM Tris (pH 7.5), 150 mM NaCl, 1 mM EDTA, 0.5% NP-40 and protease inhibitor cocktail (Roche)]. Lysates were precleared with protein A agarose beads for 1 h at 4°C with rotation followed by addition of a 1:50 dilution of NeuroD1 antibody (CST 4373S) or 5 µg of normal IgG (CST 2729S) to the lysates and incubation for 2 h at 4°C with rotation, then 30 µl of protein A beads were added and further incubated overnight at 4°C with rotation. Immunoprecipitates were washed three times with lysis buffer and eluted with 2×SDS loading buffer for 5 min. The eluted fractions were immunoblotted with Tcf12 antibody (SCBT sc-28364 X) and NeuroD1 antibody (CST 4373S) (1:1000).

### Immunofluorescence assay

A2lox-NeuroD1 ES cells were grown on coverslips. siRNA-mediated knockdowns were performed using the Lipofectamine RNAiMAX (Invitrogen) transfection reagent. NeuroD1-mediated *in vitro* neurogenesis was induced by adding doxycycline, and cells were fixed after 48 h with 4% paraformaldehyde in PBS for 15 min at room temperature. The cells were permeabilised and simultaneously blocked with 10% goat serum and 5% FBS in PBS supplemented with 0.2% Triton X-100 for 1 h at room temperature. Subsequently, the samples were incubated with β-III tubulin (Tuj1) (Abcam ab18207) overnight at 4°C. The coverslips were incubated with fluorochrome-labelled secondary antibody (Thermo Fisher Scientific, A10042) for 1 h at room temperature followed by counterstaining with DAPI, mounting with Vectashield antifade mounting media and imaging with a Leica SP5 confocal laser-scanning microscope.

### Quantitative RT-PCR

Total RNA of cultured cells was prepared using TRIzol reagent (Invitrogen) and reverse–transcribed with a First Strand cDNA Synthesis kit (Fermentas). The transcripts were quantified by qPCR using SYBR Green PCR MasterMix (ABI) on a Lightcycler 840 PCR System (Roche). Mouse TBP primers were used for normalisation of RNA expression by amplifying an intergenic region for normalisation of ChIP enrichment above background. The sequences of all primers used in this study are provided in Table S1.

### Immunoblotting

The cells were lysed in lysis buffer [10 mM Tris (pH 7.5), 150 mM NaCl, 0.1 mM EDTA, 0.5% NP-40 and protease inhibitor cocktail (Roche)], and the protein concentrations were quantified using Bradford reagent (Bio-Rad). Equal input amounts (30 μg) and IP samples from the beads were boiled in 6× SDS-PAGE loading buffer, run on a polyacrylamide gel, transferred to a PVDF membrane, blocked with 5% milk and probed with the appropriate antibodies.

### *In utero* electroporation (IUE)

Plugged C57BL/6J females were purchased from Janvier Labs and E0.5 was defined as the morning on which the vaginal plug appeared. Mice were subjected to *in utero* electroporation and embryos harvested as previously described (Artegiani et al., 2012; Aprea et al., 2013). Electroporation was performed using pDSV vector as previously described (Pataskar et al., 2016a) encoding for either Tcf12-shRNA or a scramble sequence. In brief, plasmids were prepped using an EndoFree Plasmid Maxi kit (Qiagen) and plasmid concentration adjusted to 1.5 μg/μl. Pregnant mice were anaesthetised with isofluorane at E13.5 and ∼1.5 μl of plasmid solution was injected into the ventricle of embryonic brains. The plasmids were electroporated into the dorsal ventricular zone with nine pulses of 30 V, 50 ms each at 1 s interval delivered through platinum electrodes using a BTX–830 electroporator (Genetronics). Mice were administered a single dose of BrdU 24 h after electroporation, embryos were collected either 1 or 3 days later, and tissue was processed for analyses as described previously (Artegiani et al., 2012; Pataskar et al., 2016a).

### Short hairpin RNA against Tcf12

A pair of 59-nt long oligonucleotides (5′-CCGGGAAGGCCTTGGCATCTATTTACTCGAGTAAATAGATGCCAAGGCCTTCTTTTTG-3′ and 5′-CTTCCGGAAGGCCTTGGCATCTATTTATAAATAGATGCCAAGGCCTTCGGAAGAAAAACTTAA-3′), encoding a 21 nt shRNA against mouse Tcf12 were designed. Additional AgeI and EcoRI restriction sites were incorporated in the sequences to facilitate cloning. A BLAST search was performed using the National Center for Biotechnology Information (NCBI) Expressed Sequence Tags database to confirm that the shRNA construct specifically targeted mouse Tcf12. A scrambled shRNA sequence (TTCTCCGAACGTGTCACGT), exhibiting no homology to the mouse sequence database, was employed as a negative control. The oligonucleotides were annealed and cloned into the IUE vector.

### Immunohistochemistry

Brains were fixed in 4% paraformaldehyde in 0.1 M phosphate buffer (PFA, pH 7.4) overnight at 4°C, cryoprotected in 30% sucrose and cryosectioned (10 µm). Immunohistochemistry was performed as previously described (Lange et al., 2009) using anti-GFP (1:400, Rockland, 600-301-215), anti-BrdU (1:200, Abcam, ab152095), anti-Tbr2 (1:500, Abcam, ab23345) and anti-βIII-Tub (1:1000, Sigma, T2200) antibodies with DAPI used to counterstain nuclei. Exposure to 2 M HCl for 20 min was used to reveal BrdU incorporation. Sections were imaged using an automated microscope (ApoTome; Carl Zeiss). Pictures were digitally assembled using Axiovision software (Carl Zeiss) and composites analysed using Adobe or Affinity. Targeted cells after *in utero* electroporation were identified based on GFP immunoreactivity and the relevant region of the lateral cortex was selected as a cortical column with boundaries perpendicular to the apical border. Cortical layers of the VZ, SVZ, IZ and CP were identified upon Tbr2 immunoreactivity, which defines the SVZ, and by the characteristic low density of DAPI^+^ nuclei in the IZ. The number of cells positive for any given marker was finally calculated as a proportion of the originally targeted cell pool (GFP^+^, defined as 100%).

### Chromatin immunoprecipitation

A chromatin immunoprecipitation (ChIP) assay was performed as described previously (Pataskar et al., 2016a). Briefly, A2lox-NeuroD1 cells were cross-linked in a medium containing 1% formaldehyde for 10 min at room temperature, neutralised with 125 mM glycine, scraped off and rinsed twice with 10 ml of ice-cold 1× PBS. The cells were pelleted by centrifugation for 7 min at 4°C at 600 ***g***. The pellets were resuspended in 10 ml of buffer L1 [50 mM HEPES KOH (pH 7.5), 140 mM NaCl, 1 mM EDTA (pH 8.0), 10% glycerol, 5% NP-40 and 0.25% Triton X-100] and incubated at 4°C for 10 min. This step was followed by centrifugation for 5 min at 4°C at 1300 ***g***. The pellet was resuspended in 10 ml of buffer L2 [200 mM NaCl, 1 mM EDTA (pH 8.0), 0.5 mM EGTA (pH 8.0) and 10 mM Tris (pH 8.0)] and incubated at room temperature for 10 min, followed by centrifugation for 5 min at 4°C at 1300 ***g***. The pellet was resuspended in buffer L3 [1 mM EDTA (pH 8.0), 0.5 mM EGTA (pH 8.0), 10 mM Tris (pH 8.0), 100 mM NaCl, 0.1% Na-deoxycholate and 0.17 mM N-lauroyl sarcosine] containing protease inhibitors, sonicated using a Bioruptor Plus (Diagenode) and incubated overnight at 4°C. After clearing the cellular debris by spinning at 14,000 ***g*** for 10 min at 4°C, 60 μg of chromatin was incubated overnight at 4°C with 5 µg of H3K27ac antibody (Abcam, ab-4729), HA (Abcam, ab9110) or Tcf12 antibody (SCBT, sc-28364 X) after 1 h of preclearing. The mixture was then incubated with 40 μl of protein A- or G-Sepharose beads that had been preblocked with tRNA and BSA for 3 h at 4°C. The beads were washed twice with 1 ml of buffer L3 and once with 1 ml of DOC buffer [10 mM Tris (pH 8.0), 0.25 M LiCl, 0.5% NP-40, 0.5% Na-deoxycholate and 1 mM EDTA], and the bound chromatin was eluted in 1% SDS/0.1 M NaHCO_3_. Next, treatment with RNase A (0.2 mg/ml) was performed for 30 min at 37°C followed by treatment with proteinase K (50 μg/ml) for 2.5 h at 55°C. The cross-linking was reversed at 65°C overnight with gentle shaking. The DNA was purified by phenol-chloroform extraction followed by ethanol precipitation and was recovered in 40 μl of TE buffer.

### ChIP Re-ChIP assay

ChIP Re-ChIP elutions were performed as mentioned in a previous study (Truax and Greer, 2012). First, we performed the NeuroD1 ChIP as mentioned above. NeuroD1-bound chromatin fractions were eluted by incubating the beads at 37°C with 30 µl Re-ChIP elution buffer [1× TE, 2% SDS and 15 mM DTT supplemented with protease inhibitor (Roche)] for 30 min. The eluted fraction was diluted 20 times with L3 buffer and a second immunoprecipitation with Tcf12 antibody (SCBT sc-28364 X) was carried out overnight at 4°C. The beads were washed twice with 1 ml of buffer L3 and once with 1 ml of DOC buffer, and the bound chromatin was eluted in 1% SDS/0.1 M NaHCO_3_. Next, treatment with RNase A (0.2 mg/ml) was carried out for 30 min at 37°C followed by treatment with proteinase K (50 μg/ml) for 2.5 h at 55°C. The cross-linking was reversed at 65°C overnight with gentle shaking. The DNA was purified by phenol-chloroform extraction followed by ethanol precipitation and was recovered in 40 μl of TE buffer.

### ChIP-seq analysis

ChIP-sequencing output in fastq format was subjected to stringent quality control employing FASTQC parameters. Bowtie2 was used with local parameter for soft clipping of reads and align the reads to the mouse mm10 genome with annotations from UCSC. Aligned sam files were sorted, indexed and converted to the bam format using SAMTOOLS v0.1.19. Peak calling was performed using MACS2 and taking Input as a control (Zhang et al., 2008). Peaks were merged from all samples as well as replicates to retain all the regions of interest for differential occupancy. qCount from QuasR was used to count reads from all samples in the merged peak regions. Reads were normalised for library size and enrichment was calculated using pseudocount 8. qExport from QuasR generated the bigwig files for visualisation at the genome browser.

### Bulk and single cell RNA-seq analysis

Bulk RNA-seq datasets were derived from our previous study (GSE65072). Fasta files were aligned to the mouse mm10 genome with UCSC annotations using TopHat v2.0.8 (Trapnell et al., 2009). Only uniquely mapped reads were retained for further analysis. SAMTOOLS v0.1.19 (Li et al., 2009) was used to convert the BAM output to a SAM format and to sort the BAM file. The read counts per gene were calculated using the HTSeq program v0.5.4p1 (Anders et al., 2015). The DESeq2 package (Love et al., 2014) was used to generate normalised read counts and perform differential gene expression analysis. Single cell RNA-seq data for E14.5 cortex was derived from GSE123335. Counts matrix was used to preprocess and analyse the data using Seurat v4. Monocle3 (Trapnell et al., 2014) was used for pseudotime trajectory analysis.

### Motif analysis

Motif analysis for transcriptionally induced as well as non-induced sites was performed through HOMER (Hypergeometric Optimization of Motif EnRichment) (Heinz et al., 2010; Bailey et al., 2015), which is a suite of tools for Motif Discovery and next-gen sequencing analysis. findmotifsGenome.pl tool from HOMER was used to predict the motifs and annotatePeaks.pl was used to annotate the genomic locations to the nearest genes. We have performed differential motif enrichment using Centrimo tool from MEME-ChIP suite (Bailey et al., 2015). Input primary sequences were scanned against the JASPAR database to find the enriched motifs and then Centrimo was used for motif profile enrichment with a maximum width of 200 and score ⩾5.

### ISMARA (Integrated system for motif activity response analysis)

ISMARA (Balwierz et al., 2014) is a tool to identify and model signals in the form of regulatory factors that are active in a set of samples at different time points. ISMARA focuses solely on predicted transcription factor-binding sites in proximal promoters and ignores the effects of distal enhancers. It can model the differentially active set of factors from RNA-seq or ChIP-seq data. We have used ISMARA to predict the activity of significant targets after NeuroD1 induction through H3K27ac ChIP data. We provided the H3K27ac data at 0 h (absence of NeuroD1), 12 h (+NeuroD1) and 48 h (+NeuroD1).

### Characterisation of induced and non-induced genomic site

We employed DNAShapeR (Chiu et al., 2016) to extract structure-based features of given genomic sequences. DNAshapeR scans for structural features that include information of propeller twist, major and minor groove width, and roll of nucleotides, etc. These features define the biophysical nature of the genomics location and provide information on surface accessibility and depth for binding to different factors in the vicinity.

## Supplementary Material

Supplementary information

Reviewer comments
