# Supplementary material for: Tcf12 and NeuroD1 cooperatively drive neuronal migration during cortical development
Source: Development. 2022 Feb 11;149(3):dev200250. doi: 10.1242/dev.200250 (PMC8918803; doi:10.1242/dev.200250)
Supplement: Supplementary information [file develop-149-200250-s1.pdf]

A.

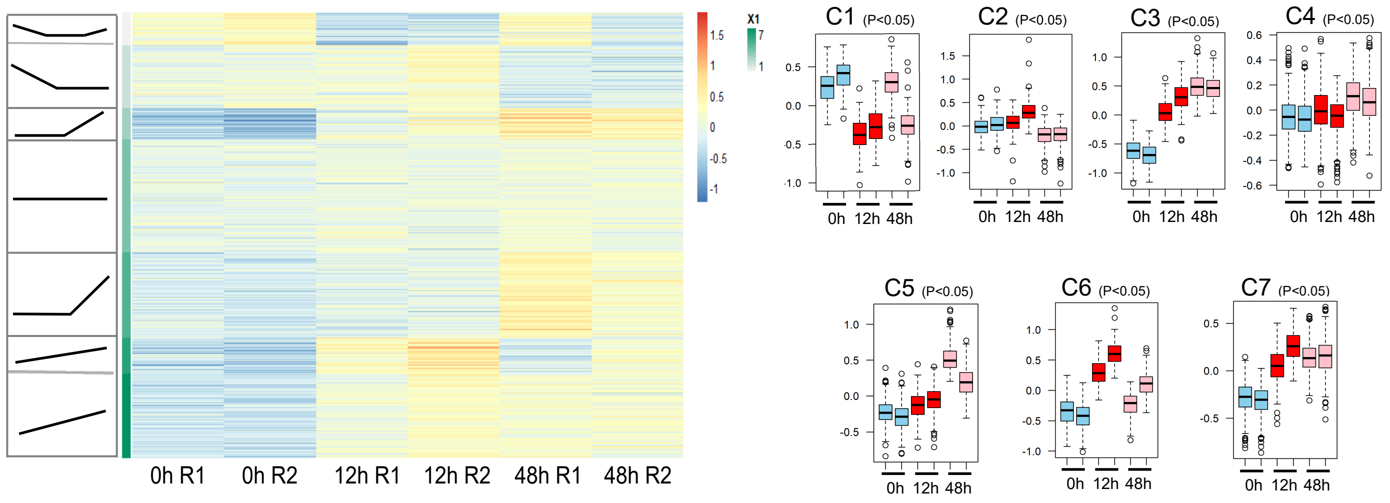

B.

Top enriched Motifs at induced sites

Rank	Motifs	Name	p-val	% of targets
1		NeuroD1	1e-567	88.05
2		Atoh1	1e-519	92.11
3		BHLHA15	1e-510	96.42
4		NeuroG2	1e-468	94.74
5		TCF4	1e-463	95.46
6		Twist2	1e-440	97.25
7		Olig2	1e-425	95.70
8		Ascl1	1e-307	84.35
9		Ptf1a	1e-269	93.79
10		Tcf12	1e-204	77.90

Top enriched Motifs at non-induced sites

Rank	Motifs	Name	p-val	% of targets
1		NeuroD1	1e-316	83.21
2		Atoh1	1e-311	90.08
3		BHLHA15	1e-280	91.79
4		NeuroG2	1e-275	92.18
5		TCF4	1e-262	91.22
6		Olig2	1e-258	94.08
7		Twist2	1e-252	93.89
8		Ascl1	1e-172	81.68
9		Tcf21	1e-150	63.74
10		Ptf1a	1e-144	90.27

C.

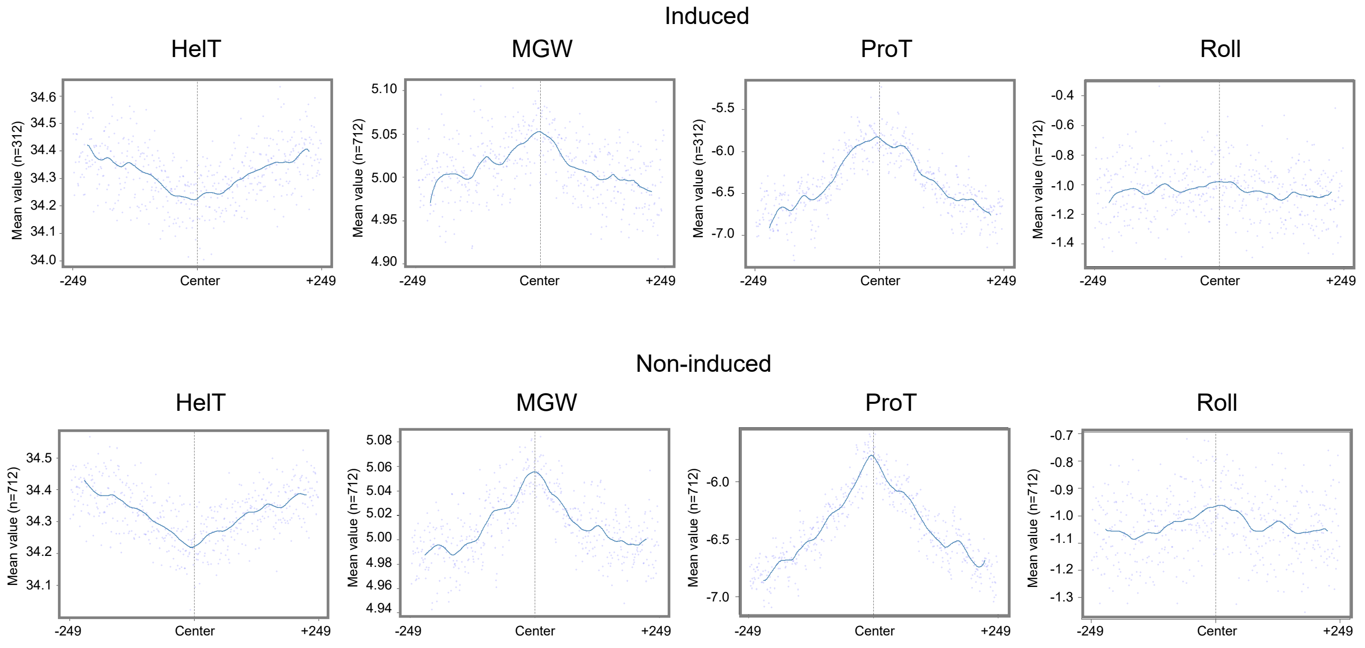

Fig. S1. A. Heat map showing the dynamics of H3K27ac levels at NeuroD1 binding sites. Boxplots on right side show the average enrichment in clusters (C1 to C7). R1 and R2 are replicates here. **B.** Motif enrichment at transcriptionally active and inactive sites. **C.** Different DNAshape biophysical features at transcriptionally induced and non-induced sites; HelT: Helix Twist, ProT: Propeller Twist, MGW: Minor groove width.

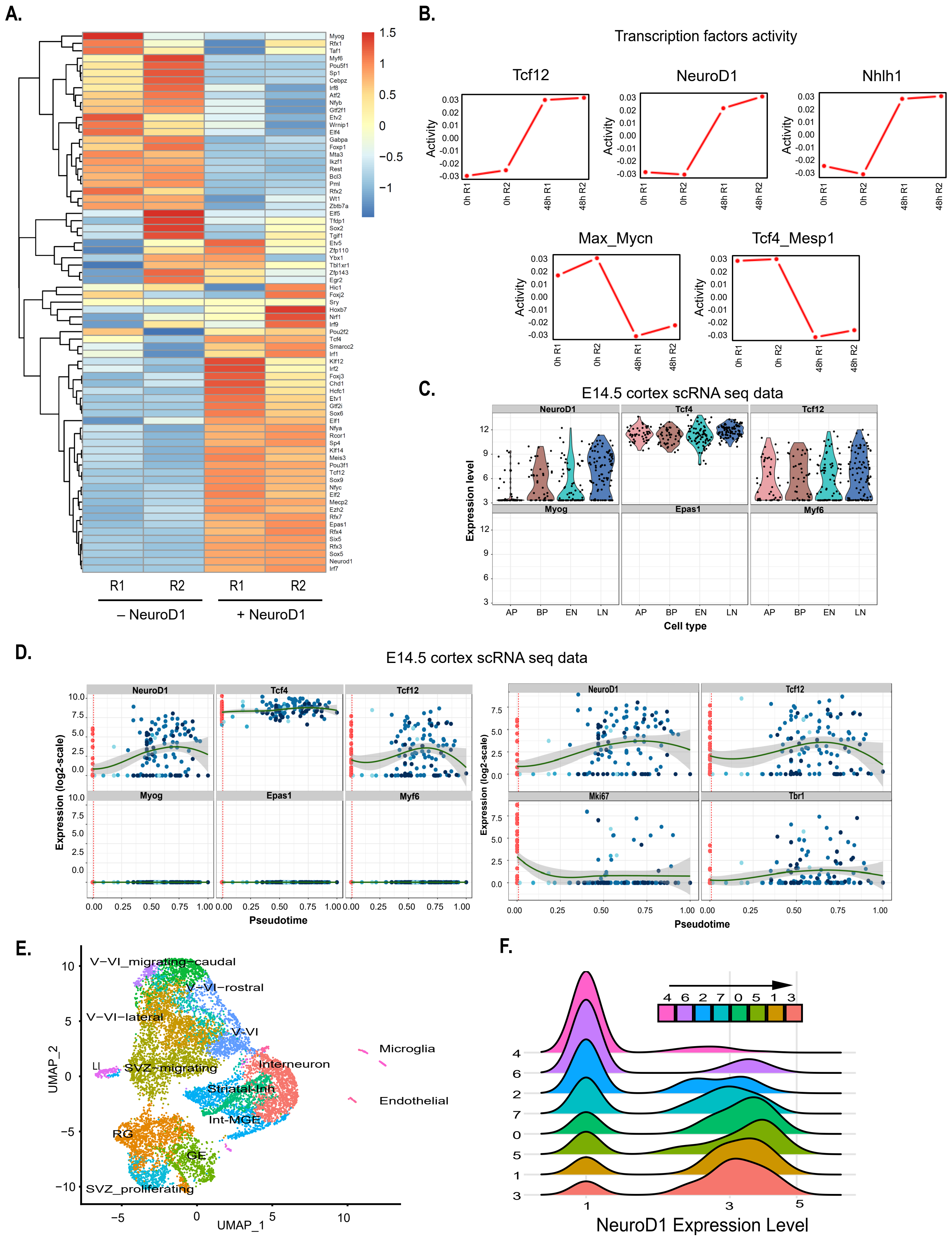

Fig. S2. A. Overlap of upregulated motifs predicted from ISMARA with bHLH transcription factors; **B.** Activity profiles for selected motifs from overlap of ISMARA motifs and bHLH factors from RNA-seq data; Max, & Mycn as well as Tcf4 & Mesp1 are independent TFs but grouped as they showed similar activity; **C**. Violin plot from scRNA-seq data shows the expression of selected TFs in different brain cell types at embryonic stage E14.5. **D.** Pseudotime expression pattern of selected TFs from the scRNA-seq data of the cortical layers at embryonic stage E14.5. **E.** UMAP plot showing cell type clusters in E14.5 cortex. **F.** SVZ migrating cell subpopulations arranged by increasing NeuroD1 expression (least in cluster 4 and highest in cluster 3, indicated on X axis).

Development • Supplementary information

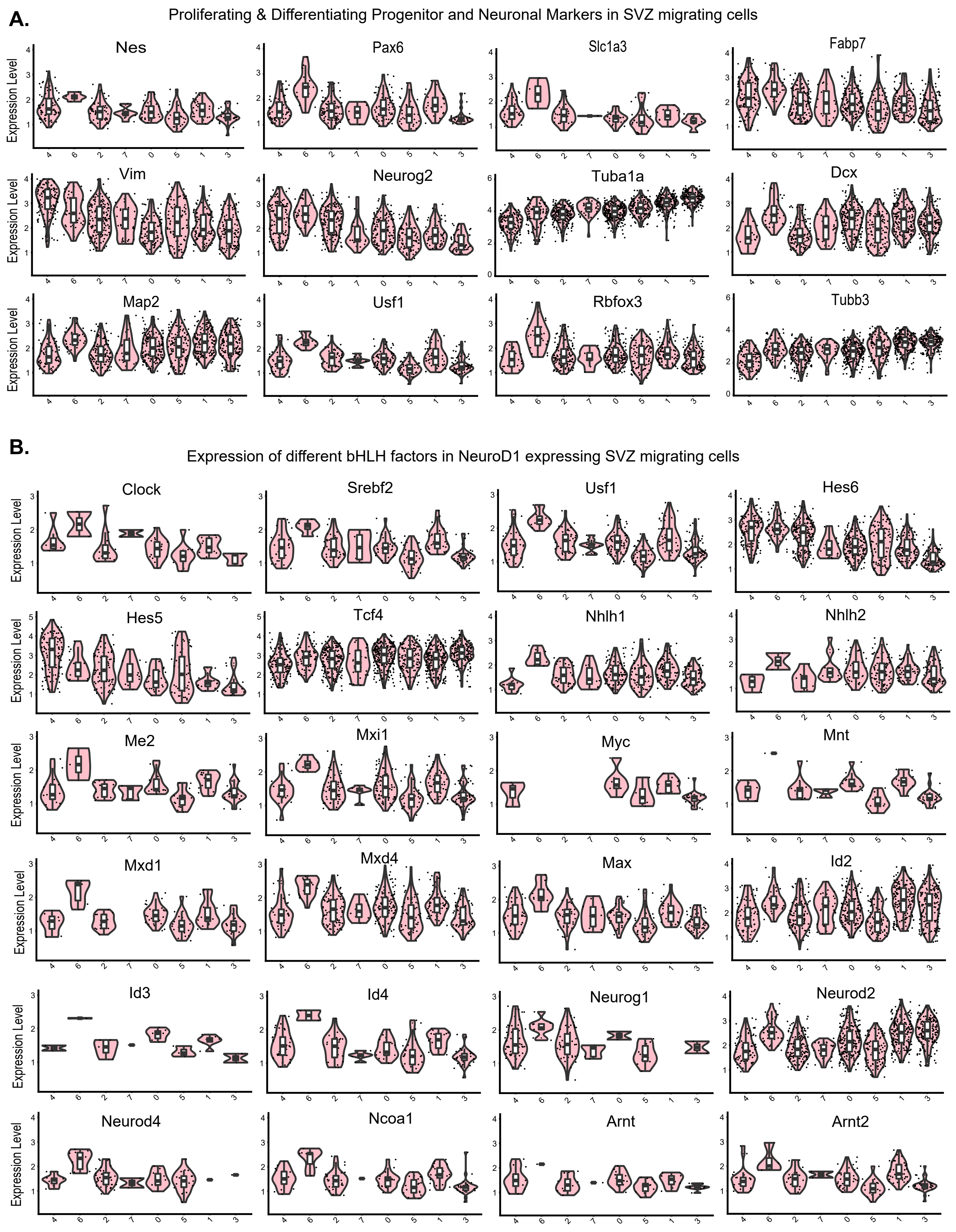

Fig. S3. A. Expression of Progenitor, Neurogenic and Neuronal markers in SVZ migrating cell populations; X axis indicates cluster numbers arranged by increasing NeuroD1 expression levels. **B.** Expression of different bHLH factors in SVZ migrating cells; X axis indicates cluster numbers arranged by increasing NeuroD1 expression levels.

Development • Supplementary information

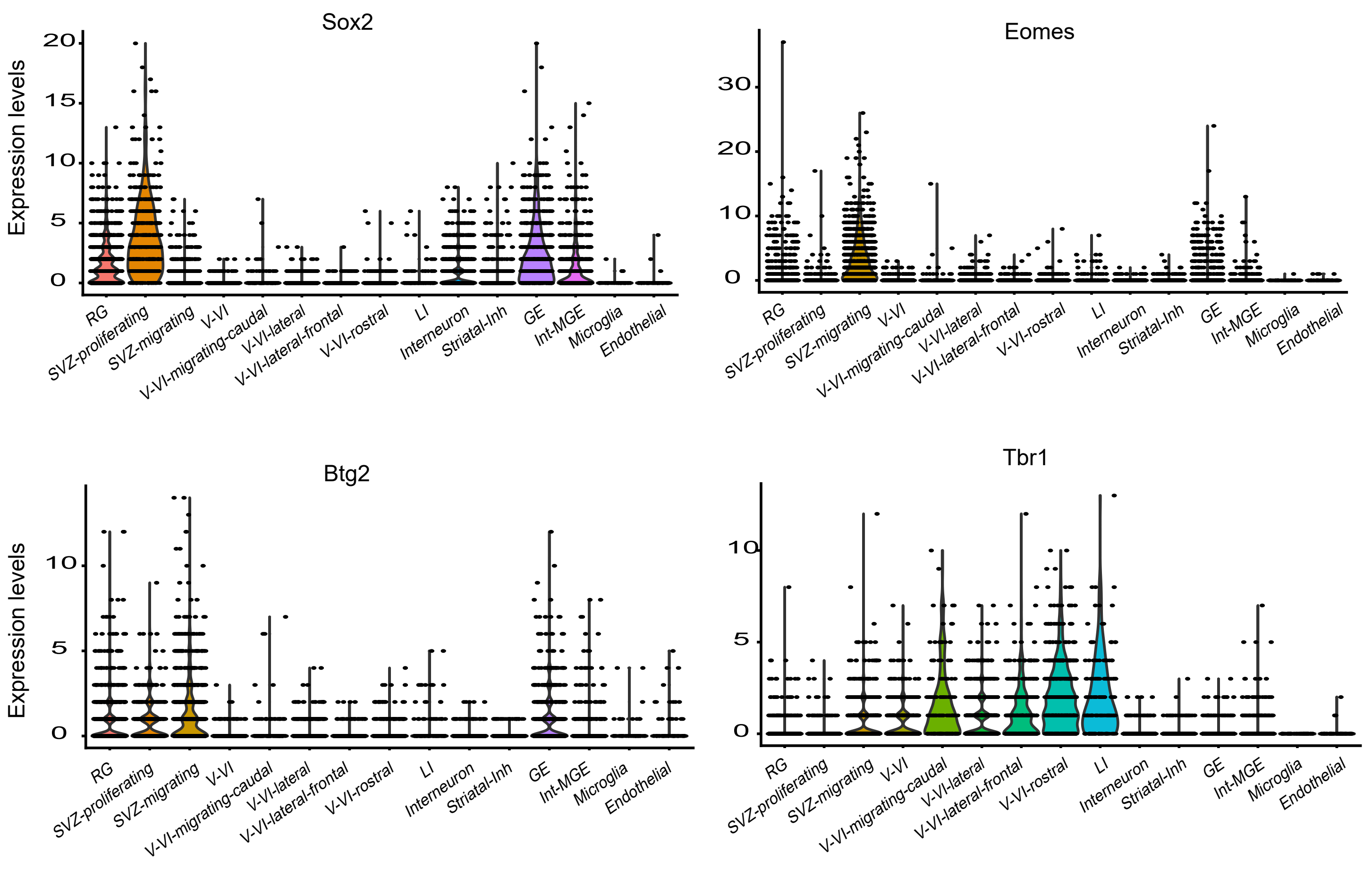

Fig. S4. Expression of progenitor and neuronal markers in E14.5 cortical cells. Proliferating cells have increased Sox2 levels, differentiating cells have higher Btg2 and Eomes while neurons have higher Tbr1.

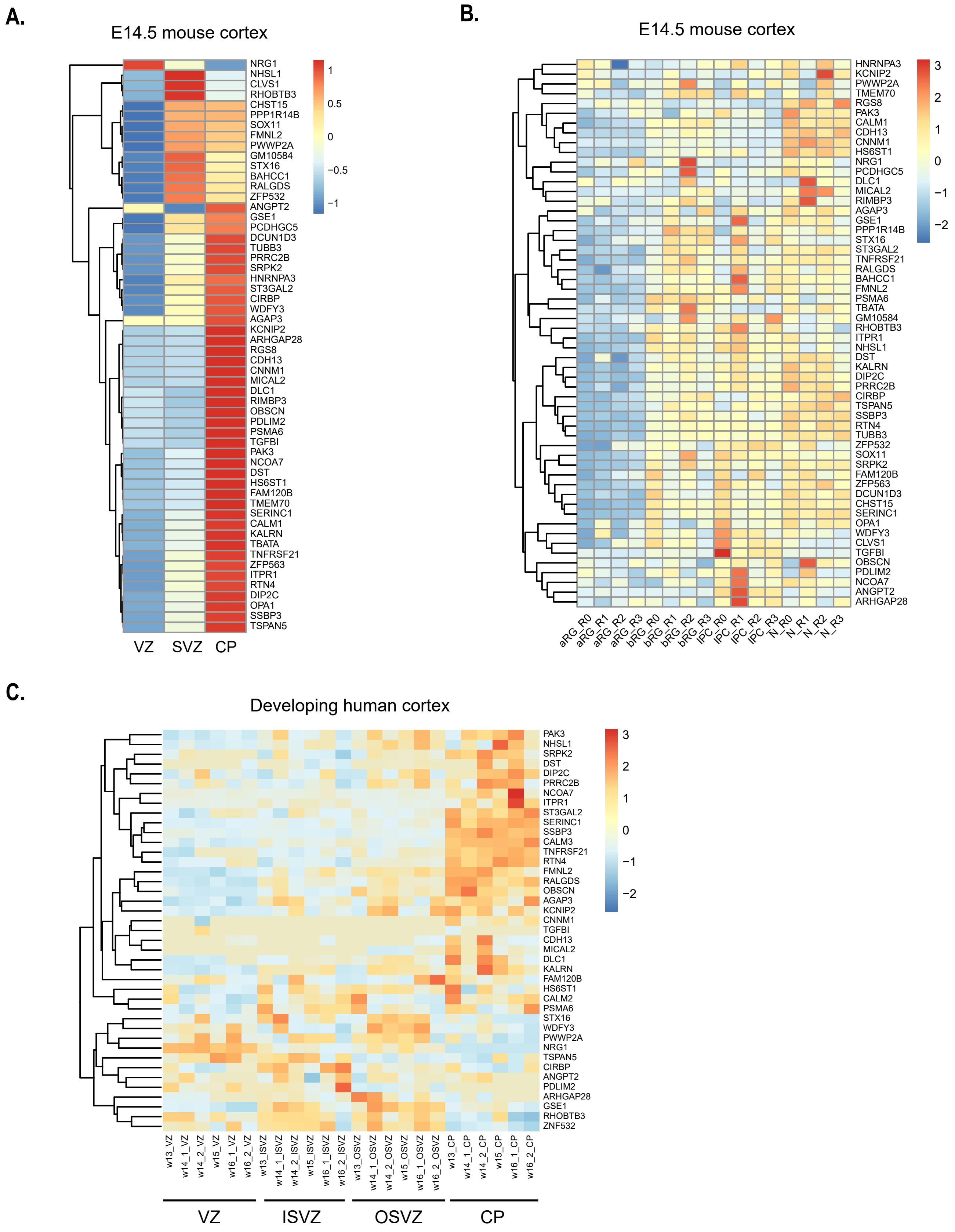

Fig. S5. A. Heatmap showing the expression of NeuroD1-Tcf12 coregulated genes in VZ, SVZ and CP of E14.5 developing mouse cortex. **B.** Heatmap showing the expression of NeuroD1-Tcf12 coregulated genes in aRG, bRG, IPC and in neurons of E14.5 developing mouse cortex. **C.** Heatmap showing the expression of NeuroD1-Tcf12 coregulated genes in VZ, ISVZ, OSVZ and CP from different gestation weeks of human brain development.

Development • Supplementary information

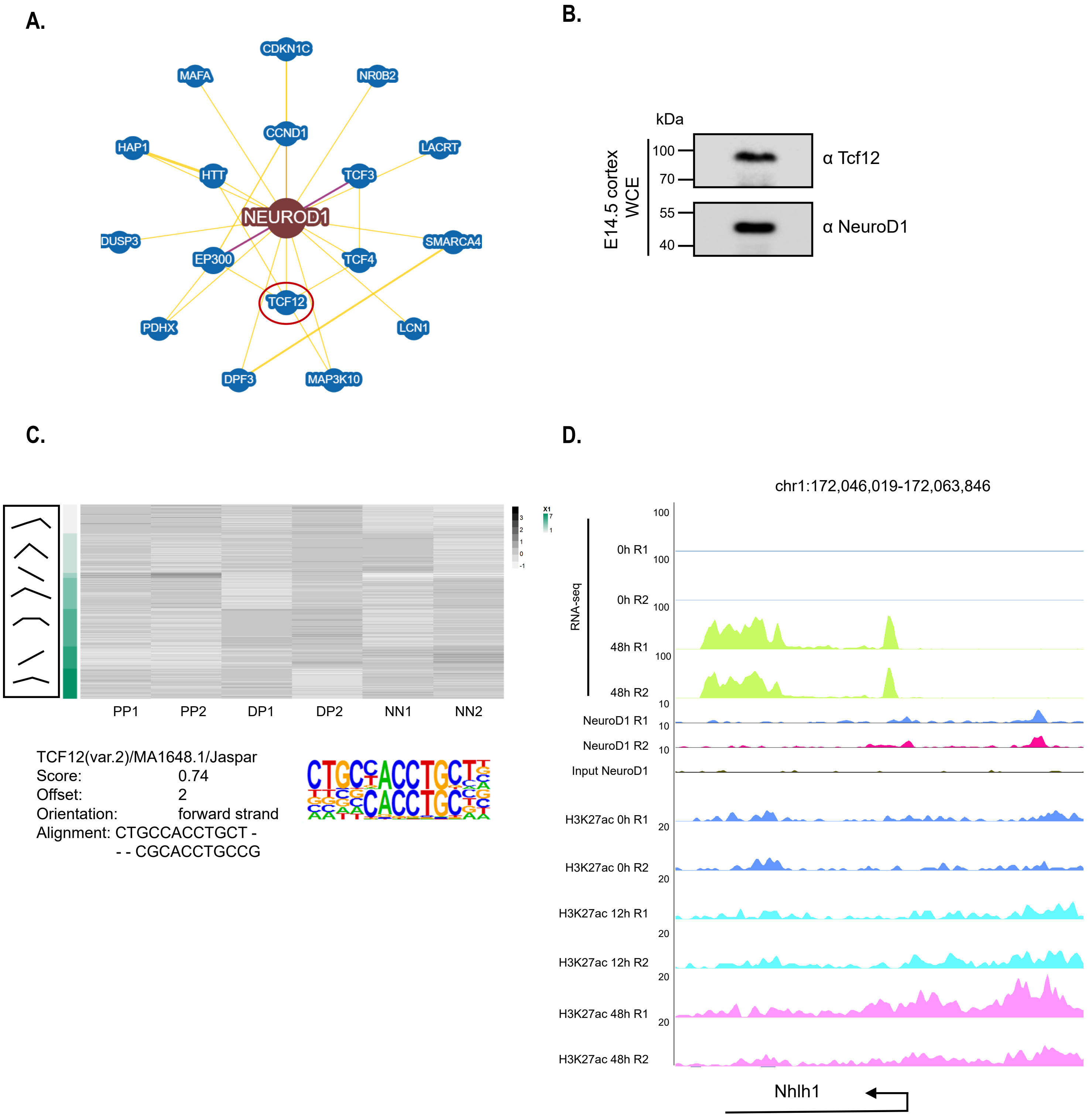

Fig. S6. A. BioGRID online tool analysis showing the interaction network of NeuroD1, which contains Tcf12 and other bHLH TFs. **B.** Immunoblot showing the expression levels of Tcf12 and NeuroD1 in E14.5 developing mouse cortex. **C.** Heat map (top) showing the chromatin accessibility dynamics at NeuroD1 binding sites in proliferative progenitors (PP), differentiating progenitors (DP) and in neurons (NN) and (bottom) Tcf12 motif alignment and de-novo motif score at the sites gaining activity in differentiating progenitors and neurons. **D.** Regulatory genomic sites of Nhlh1, co-enriched NeuroD1, also showing the NeuroD1 transcriptome tracks and H3K27ac enrichment at these sites.

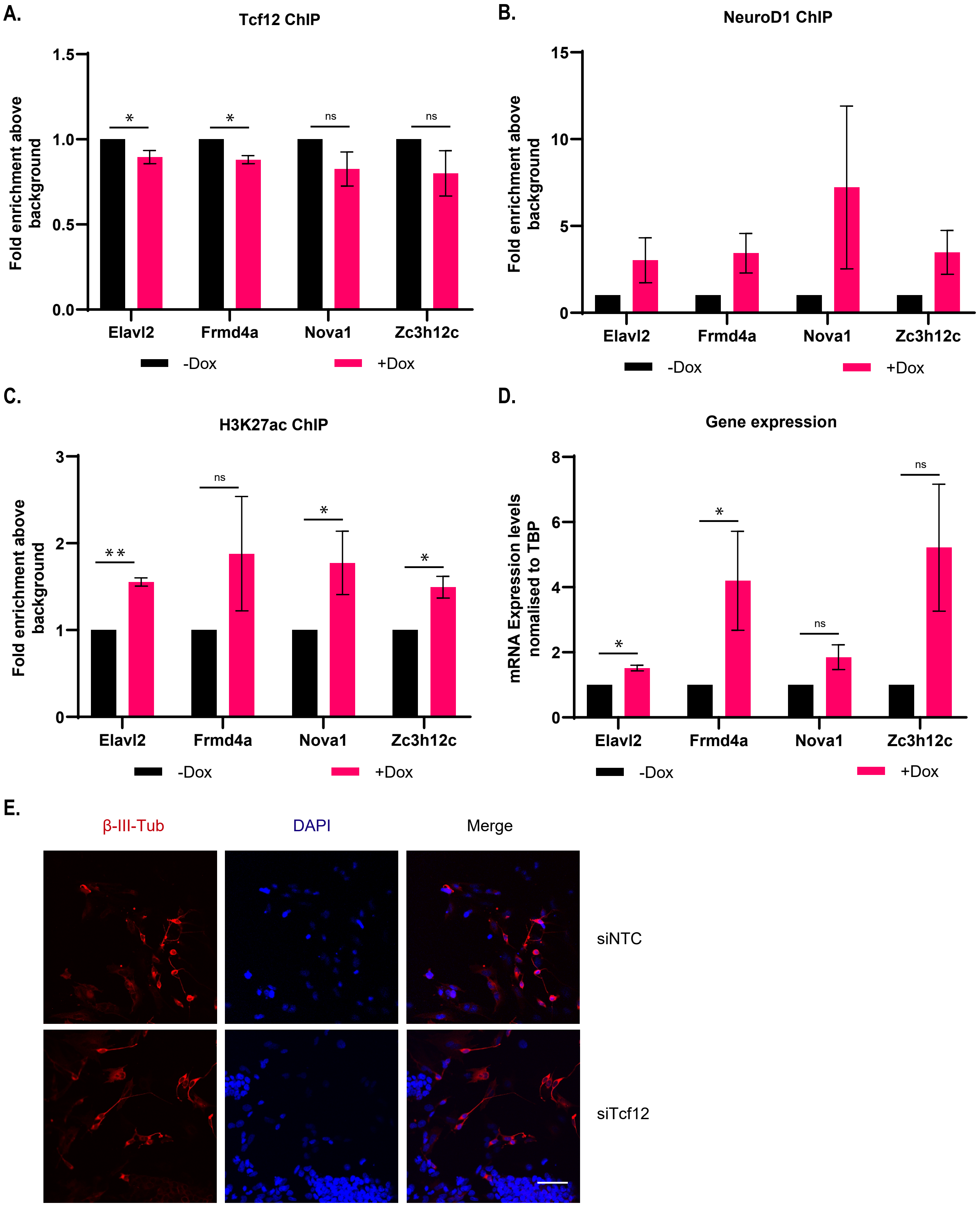

Fig. S7. A. ChIP qPCR results showing Tcf12 is not enriched at its non-targets confirming not all the NeuroD1 target genes were co-bound by Tcf12. **B**. ChIP qPCR results showing NeuroD1 binding at Tcf12 non-target genes, and these genes gain active chromatin **(C)** and gene expression **(D)** during in vitro neurogenesis. **E.** Immunofluorescence images showing knockdown of Tcf12 did not affected NeuroD1 induced *in vitro* neurogenesis in ES cell cultures. Statistical significance was calculated using two tailed student's t-test. *p < 0.05, **p < 0.01, ***p < 0.001.

Table S1. Primer sequences

Click here to download Table S1

Development • Supplementary information
