# Supplementary material for: Tcf12 and NeuroD1 cooperatively drive neuronal migration during cortical development
Source: Development. 2022 Feb 11;149(3):dev200250. doi: 10.1242/dev.200250 (PMC8918803; doi:10.1242/dev.200250)
Supplement: Reviewer comments [file develop-149-200250_review_history.pdf]

# Tcf12 and NeuroD1 cooperatively drive neuronal migration during cortical development

Aditi Singh, Arun Mahesh, Beatriz Toledo, Florian Noack, Federico Calegari and Vijay K. Tiwari

DOI: 10.1242/dev.200250

**Editor**: James Briscoe

**Review timeline**

| | |
|---|---|
| Original submission: | 6 October 2021 |
| Editorial decision: | 10 December 2021 |
| First revision received: | 21 December 2021 |
| Editorial decision: | 23 December 2021 |
| Second revision received: | 23 December 2021 |
| Accepted: | 31 December 2021 |


---

**Original submission**

First decision letter

MS ID#: DEVELOP/2021/200250

MS TITLE: Tcf12 and NeuroD1 cooperatively drive neuronal migration during cortical development

AUTHORS: Aditi Singh, Arun Mahesh, Beatriz Toledo, Florian Noack, Federico Calegari, and Vijay K Tiwari

I apologise for the long time it has taken to consider your paper. Although we contacted the three original referees used by Review Commons, I'm afraid we have only been able to receive comments on your revision from one them. I have used these and the original referees comments, together with your response, to reach a decision. The referees' comments are appended below, or you can access them online: please go to BenchPress and click on the 'Manuscripts with Decisions' queue in the Author Area.

I am pleased to say that the overall evaluation is positive and we would like to publish a revised manuscript in Development. The reviewer's comments on your manuscript make several helpful suggestions to increase the clarity of your study that I would encourage you to address. I also appreciate the revisions you made to your study in response to the original round of refereeing. The one issue remaining from these that I would encourage you to consider is the data concerning the co-expression of NeuroD1 and Tcf12 in the SVZ. I understand that limitations with reagents has made co-expression difficult to confirm. I suggest you make clear this caveat in your Discussion.

Please attend to all of the reviewers' comments in your revised manuscript and detail them in your point-by-point response. If you do not agree with any of their criticisms or suggestions explain clearly why this is so.

We are aware that you may currently be unable to access the lab to undertake experimental revisions. If it would be helpful, we encourage you to contact us to discuss your revision in greater detail. Please send us a point-by-point response indicating where you are able to address concerns raised (either experimentally or by changes to the text) and where you will not be able to do so

© 2022. Published by The Company of Biologists under the terms of the Creative Commons Attribution License (https://creativecommons.org/licenses/by/4.0/).

within the normal timeframe of a revision. We will then provide further guidance. Please also note that we are happy to extend revision timeframes as necessary.

Reviewer 1

*Advance summary and potential significance to field*

It is unknown how NeuroD1 (a bHLH TF) is able to coordinate several milestones of corticogenesis independently (transition from AP-BP, neurogenesis, migration, maturation and survival of newborn neurons). In this paper the authors describe how heterodimerization of NeuroD1 and Tcf12 affects new-born-neuronal migration through a gain of active chromatin sites and expression of genes involved in cell migration. Previous work (also from the involved groups) described NeuroD1 as a pioneer transcription factor that imposes euchromatic states leading to pro-neurogenic gene expression. Thus, it has been speculated that distinct heterodimerization may coordinate several process across different cell types. The data shown here add novel insights how Tcf12 may achieve stage-specific functions. The experimental design is straightforward. The results presented increase the knowledge how bHLH transcription and E-box transcription factor heterodimerization may lead to cell-stage specific effects. Conceptually, this is not entirely novel and the authors have already cited important previous work (e.g., Fischer et al., 2014 Neural Dev PMID: 25352248 and Le Dreau et al., 2018 eLife PMID: 30095408). However, the data will be of interest to the field and will advance (incrementally) our understanding of brain development and I will support itÂ’s publication with some minor changes. A lot of extra work has been added since it was reviewed before (extra IUE, scRNA-seq).

*Comments for the author*

- Comments on abstract: The last sentence of the abstract is not supported by the data “Our study suggests that the combinatorial heterodimerization of distinct bHLH TFs enables synchronizing distinct events during tissue formation, which may be relevant beyond the brain and also apply to other organ systems.” In the scope of this paper they are only discussing NeuroD1 and Tcf12, therefore making conclusions regarding general heterodimerization of distinct bHLH TFs is too far from their conclusions.
- NeuroD1 bound target sites exhibit distinct DNA shape features and motif enrichment for other bHLH TFs including Tcf12. Increased transcription correlates with high local density of adjacent bHLH TF motifs, mostly, Tcf12. The authors use mouse NSCs with selective induction of NeuroD1 leading to neuronal differentiation and perform ChIP-seq for H3K27ac, 12 and 48h after induction and detect 7 behaviors/clusters of genomic regions. The authors added boxplots and test statistics to compare the replicates from the ChIP-seq, as suggested. Changes in DNA shape are shown and clarified.
o In the text referring to Fig 1D, saying “only a fraction of these was transcriptionally induced (694)” may not be fully accurate, considering that half of the sites where NeuroD1 is bound seem to be transcriptionally activated. We suggest to rephrase.
o Fig 1E is not mentioned in the text but should be added since it refers to the biophysical properties of DNA binding of NeuroD1.
- Activity and expression levels of Tcf12 were high in cells with induced levels of NeuroD1. The authors show convincingly that NeuroD1 and Tcf12 behave similarly with ISMARA modelling and that Tcf12 and NeuroD1 are enriched in SVZ through RNAseq and Allen Brain Atlas images. They further confirm this with newly provided scRNA-seq.
o Fig 3A. The point of showing all cell clusters in a graph like that is somewhat unclear. It would be more explanatory to show maps of the clusters with intensities of NeuroD1 and Tcf12 and see that they are indeed enriched in migrating neurons.
o Fig 3B. The dots of the pseudotime data should be enlarged and the name of the population from the cluster should be included.
o The colors in Fig3C are not helpful if the map of clusters is not in the main figure.
o Fig S5C. A title should be put on the graph.

- KO-Tcf12 impairs neuronal migration but does not affect cell-fate specification (mostly Fig5). They used IUE to show that knock-down of Tcf12 for 2 and 4 days causes defects in cell migration but no changes in cell fate (same proportion of all cell types), which is an interesting and

© 2022. Published by The Company of Biologists under the terms of the Creative Commons Attribution License (https://creativecommons.org/licenses/by/4.0/).

convincing result. These data represent a large amount of extra work. However, Figure 5 should be revised and improved.
o Fig 5. The dots are weirdly stretched out. Indeed, only data-points and no bar graphs should be shown. The scale in graphs showing percentages should be harmonized, showing always 0-100% and not changing it every time. The authors should include the statistics for ALL the comparisons, not just the significant ones.
o It is somewhat unclear which populations were chosen to calculate percentages. (B) Televance of counting the number of GFP+ cells within the Tbr2+ population is unclear: rather GFP+Tbr2+/Tbr2+ or GFP+Tbr2+/GFP+ should be analyzed. The provided phenotyping is OK but considering that there are good markers for both APs and neurons (plus one extra channel “available”) this could be improved.
o (C) In the bin classification, it shouldbe stated explicitly: GFP+BrdU+ reduced in bin3-5 and so on, not just vague upper or lower bins.
o (F) It seems obvious “by eye”, but the whole set of IUE experiments, requires at least the basic quantification of GFP+ cells per region.

---

**First revision**

Author response to reviewers' comments

**RESPONSES TO REVIEWERS**

**Reviewer 1 (Remarks to the Author):**

We thank the reviewer for her/his nice summary, ***“It is unknown how NeuroD1 (a bHLH TF) is able to coordinate several milestones of corticogenesis independently (transition from AP-BP, neurogenesis, migration, maturation and survival of newborn neurons). In this paper the authors describe how heterodimerization of NeuroD1 and Tcf12 affects new-born-neuronal migration through a gain of active chromatin sites and expression of genes involved in cell migration. Previous work (also from the involved groups) described NeuroD1 as a pioneer transcription factor that imposes euchromatic states leading to pro-neurogenic gene expression. Thus, it has been speculated that distinct heterodimerization may coordinate several process across different cell types. The data shown here add novel insights how Tcf12 may achieve stage-specific functions.” The reviewer also states “The results presented increase the knowledge how bHLH transcription and E-box transcription factor heterodimerization may lead to cell-stage specific effects.” and “the data will be of interest to the field and will advance our understanding of brain development”***. She/he further comments “***I will support it’s publication with some minor changes. A lot of extra work has been added since it was reviewed before (extra IUE, scRNA-seq).”***

The reviewer had some minor suggestions that we have addressed as follows:

***Comment 1)*** *Comments on abstract: The last sentence of the abstract is not supported by the data “Our study suggests that the combinatorial heterodimerization of distinct bHLH TFs enables synchronizing distinct events during tissue formation, which may be relevant beyond the brain and also apply to other organ systems.” In the scope of this paper they are only discussing NeuroD1 and Tcf12, therefore making conclusions regarding general heterodimerization of distinct bHLH TFs is too far from their conclusions.*
**Authors’ response:** We agree with the reviewer and we have now removed this text.

***Comment 2)*** *NeuroD1 bound target sites exhibit distinct DNA shape features and motif enrichment for other bHLH TFs including Tcf12. Increased transcription correlates with high local density of adjacent bHLH TF motifs, mostly, Tcf12. The authors use mouse NSCs with selective induction of NeuroD1 leading to neuronal differentiation and perform ChIP-seq for H3K27ac, 12 and 48h after induction and detect 7 behaviors/clusters of genomic regions. The authors added boxplots and test statistics to compare the replicates from the ChIP-seq, as suggested. Changes in DNA shape are shown and clarified.*

© 2022. Published by The Company of Biologists under the terms of the Creative Commons Attribution License (https://creativecommons.org/licenses/by/4.0/). 

**Authors' response:** We thank the reviewer for this positive feedback and glad these *developments are satisfactory.*

***Comment 3)*** *In the text referring to Fig 1D, saying "only a fraction of these was transcriptionally induced (694)" may not be fully accurate, considering that half of the sites where NeuroD1 is bound seem to be transcriptionally activated. We suggest to rephrase.*
**Authors' response:** We thank the reviewer for pointing this out and we have now rephrased to state that "approximately half of these were transcriptionally induced (694)" (Fig. 1D).

***Comment 4)*** *Fig 1E is not mentioned in the text but should be added since it refers to the biophysical properties of DNA binding of NeuroD1.*
**Authors' response:** We thank the reviewer for pointing this out and we have now cited Fig 1E.

***Comment 5)*** *Activity and expression levels of Tcf12 were high in cells with induced levels of NeuroD1. The authors show convincingly that NeuroD1 and Tcf12 behave similarly with ISMARA modelling and that Tcf12 and NeuroD1 are enriched in SVZ through RNAseq and Allen Brain Atlas images. They further confirm this with newly provided scRNA-seq.*
**Authors' response:** We are delighted that the reviewer finds these expanded analysis of the activity and expression of NeuroD1 and Tcf12 satisfactory. We thank the reviewer for suggesting these analyses that have significantly increased the impact of our findings.

***Comment 6)*** *Fig 3A. The point of showing all cell clusters in a graph like that is somewhat unclear. It would be more explanatory to show maps of the clusters with intensities of NeuroD1 and Tcf12 and see that they are indeed enriched in migrating neurons.*
**Authors' response:** We thank the reviewer for this suggestion and we have now updated the graph as advised. The new figure clearly shows that NeuroD1 and Tcf12 are expressed in migrating neuronal populations.

***Comment 7)*** Fig *3B. The dots of the pseudotime data should be enlarged and the name of the population from the cluster should be included.*
**Authors' response:** We thank the reviewer for this suggestion. We have now enlarged the dots in the pseudotime plot and the name of the population from the cluster is included.

***Comment 8)*** *The colors in Fig3C are not helpful if the map of clusters is not in the main figure.*
**Authors' response:** We have now updated the figure by using uniform colors in all the clusters.

***Comment 9)*** *Fig S5C. A title should be put on the graph.*
**Authors' response:** We have now put the title on the graph.

***Comment 10)*** *KO-Tcf12 impairs neuronal migration but does not affect cell-fate specification (mostly Fig5). They used IUE to show that knock-down of Tcf12 for 2 and 4 days causes defects in cell migration but no changes in cell fate (same proportion of all cell types), which is an interesting and convincing result. These data represent a large amount of extra work.*
**Authors' response:** We thank the reviewer for her/his extremely positive feedback on these findings.

***Comment 11)*** *However, Figure 5 should be revised and improved. The dots are weirdly stretched out. Indeed, only data-points and no bar graphs should be shown. The scale in graphs showing percentages should be harmonized, showing always 0-100% and not changing it every time. The authors should include the statistics for ALL the comparisons, not just the significant ones.*
**Authors' response:** We thank the reviewer for pointing this out. We have now improved the figures and kept the graph scales 0-100% uniformly in all the graphs in figure 5. We have now also added statistics for all the comparisons made in the analysis.

***Comment 12)*** *It is somewhat unclear which populations were chosen to calculate percentages. (B) Televance of counting the number of GFP+ cells within the Tbr2+ population is unclear: rather GFP+Tbr2+/Tbr2+ or GFP+Tbr2+/GFP+ should be analyzed. The provided phenotyping is OK but considering that there are good markers for both APs and neurons (plus one extra channel "available") this could be improved.*
**Authors' response:** We thank the reviewer for suggestion these new quantifications. We have now

© 2022. Published by The Company of Biologists under the terms of the Creative Commons Attribution License (https://creativecommons.org/licenses/by/4.0/).

quantified GFP+Tbr2+/Tbr2+ populations and added in these figures. These data highlight and are in line with our previous observations.

*Comment 13) (C) In the bin classification, it should be stated explicitly: GFP+BrdU+ reduced in bin3-5 and so on, not just vague upper or lower bins.*
**Authors' response:** We thank the reviewer for indicating this. We have now changed the sentence in the main text to "*Concomitantly, the proportion of GFP+ BrdU+ cells within the bins 3 to 5 were reduced*".

*Comment 14) (F) It seems obvious "by eye", but the whole set of IUE experiments, requires at least the basic quantification of GFP+ cells per region.*
**Authors' response:** We have now quantified GFP+ populations in VZ/SVZ, IZ and CP regions and provided this quantifications next to the images. These observations are in line with our previous findings. We thank the reviewer for suggesting these quantifications that has increased the impact of our results.

---

Second decision letter

MS ID#: DEVELOP/2021/200250

MS TITLE: Tcf12 and NeuroD1 cooperatively drive neuronal migration during cortical development

AUTHORS: Aditi Singh, Arun Mahesh, Beatriz Toledo, Florian Noack, Federico Calegari, and Vijay K Tiwari

As we discussed. Please upload the new version of your manuscript.

We are aware that you may currently be unable to access the lab to undertake experimental revisions. If it would be helpful, we encourage you to contact us to discuss your revision in greater detail. Please send us a point-by-point response indicating where you are able to address concerns raised (either experimentally or by changes to the text) and where you will not be able to do so within the normal timeframe of a revision. We will then provide further guidance. Please also note that we are happy to extend revision timeframes as necessary.

---

Third decision letter

MS ID#: DEVELOP/2021/200250

MS TITLE: Tcf12 and NeuroD1 cooperatively drive neuronal migration during cortical development

AUTHORS: Aditi Singh, Arun Mahesh, Beatriz Toledo, Florian Noack, Federico Calegari, and Vijay K Tiwari
ARTICLE TYPE: Research Article

I am happy to tell you that your manuscript has been accepted for publication in Development, pending our standard ethics checks.

© 2022. Published by The Company of Biologists under the terms of the Creative Commons Attribution License (https://creativecommons.org/licenses/by/4.0/).
